# Supplementary material for: Dual Role of WISP1 in maintaining glioma stem cells and tumor-supportive macrophages in glioblastoma
Source: Nat Commun. 2020 Jun 15;11:3015. doi: 10.1038/s41467-020-16827-z (PMC7295765; doi:10.1038/s41467-020-16827-z)
Supplement: Supplementary file 1 — Supplementary Information [file 41467_2020_16827_MOESM1_ESM.pdf]

# Supplementary Information

## Dual Role of WISP1 in Maintaining Glioma Stem Cells and Tumor-supportive Macrophages in Glioblastoma

Weiwei Tao et al.

**Supplementary Figure 1**

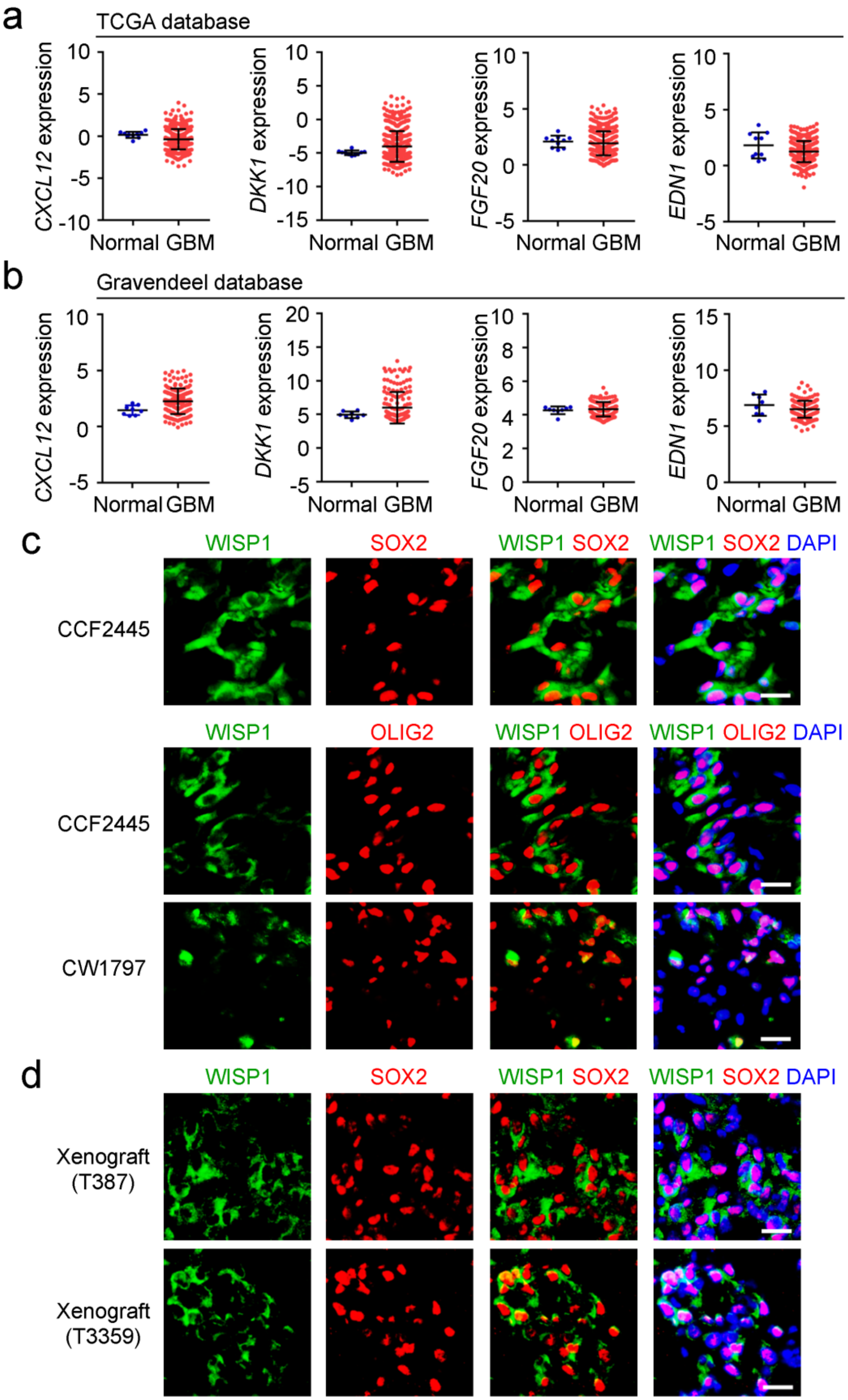

**Supplementary Figure 1. Co-expression of WISP1 with the GSC markers in GBMs.**

**a,** Gene expression of the Wnt/β-catenin-induced secretory proteins in normal human brain tissues and GBM tumor samples from the TCGA database. Normal, n=10; GBM, n=488 (*FGF20*) or 489 (*CXCL12*, *DKK1*, *EDN1*). Data are shown as means ± s.d.

**b,** Gene expression of the Wnt/β-catenin-induced secretory proteins in normal human brain tissues and GBM tumor samples from the Gravendeel database. Normal, n=8; GBM, n=159. Data are shown as means ± s.d.

**c,** Immunofluorescent staining of WISP1 (green) and the GSC marker SOX2 or OLIG2 (red) in human primary GBMs. Scale Bar, 20 μM.

**d,** Immunofluorescent staining of WISP1 (green) and the GSC marker SOX2 (red) in GSC-derived GBM xenografts. Scale Bar, 20 μM.

Source data are provided as a Source Data file.

**Supplementary Figure 2**

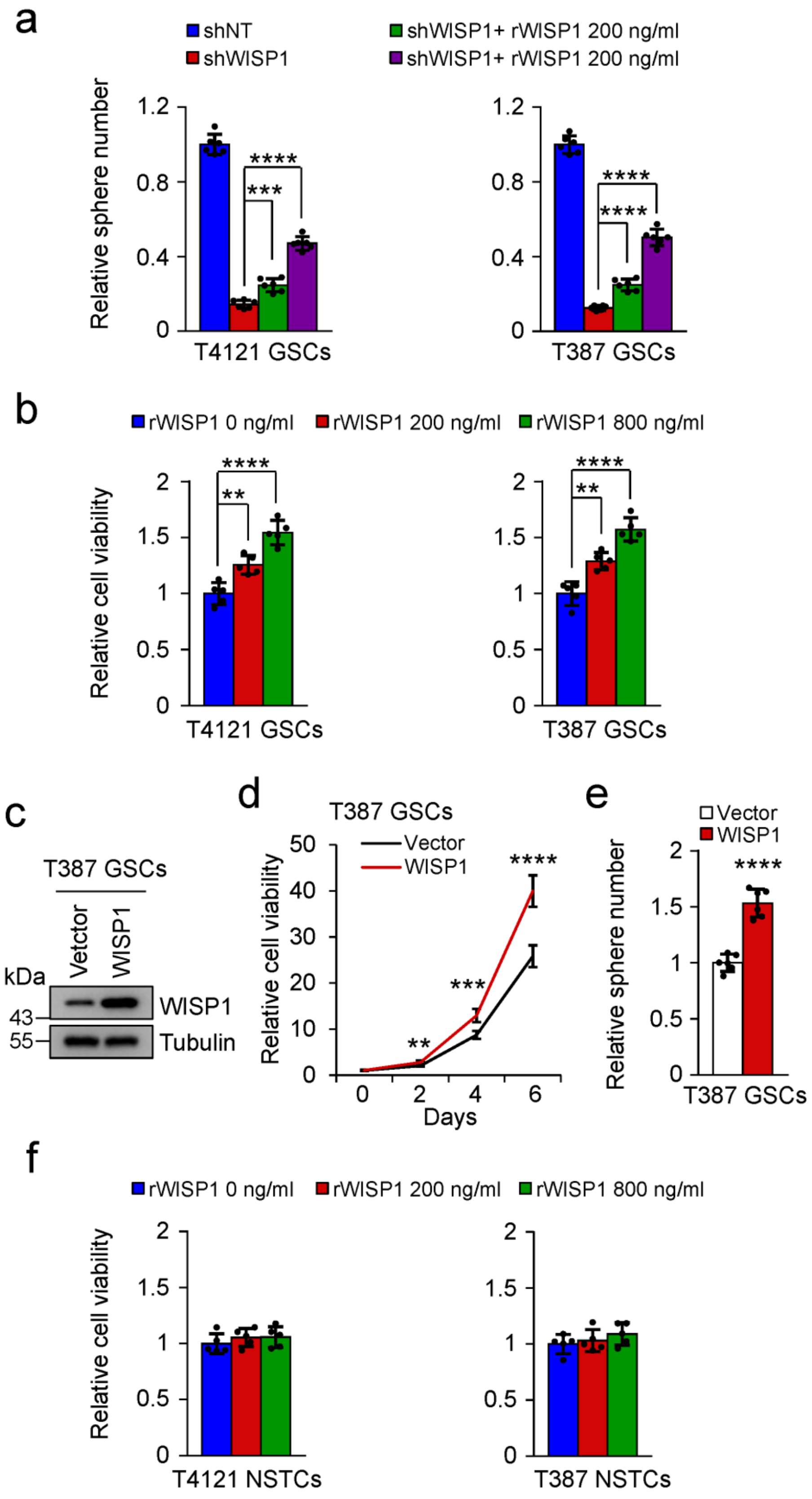

**Supplementary Figure 2. Secreted WISP1 promotes GSC growth but has little impact on NSTC growth or survival.**

**a,** Tumorsphere formation of GSCs transduced with shNT or WISP1 shRNA (shWISP1) and cultured with different dose of recombinant human WISP1 (rWISP1) protein for 4 days. n=6 biological independent cell cultures. Data are represented as means ± s.d. ***$p$=0.001, ****$p < 0.0001$, two-tailed unpaired $t$-test.

**b,** Cell vaibility of GSCs cultured with different dose of rWISP1 protein for 4 days. n=5 biological independent samples. Data are shown as means ± s.d. **$p$=0.0022 (T4121 GSCs), **$p$=0.0011 (T387 GSCs), ****$p < 0.0001$, two-tailed unpaired $t$-test.

**c,** Immunoblot analysis of WISP1 expression in T387 GSCs after transduction with WISP1 overexpression or vector control.

**d,** Cell viability assay of T387 GSCs transduced with WISP1 overexpression or vector control. n=6 biological independent cell cultures. Data are represented as means ± s.d. **$p$=0.0012, ***$p$=0.0001, ****$p < 0.0001$, two-tailed unpaired $t$-test.

**e,** Tumorsphere formation of T387 GSCs transduced with WISP1 overexpression or vector control. n=6 biological independent samples. Data are represented as means ± s.d. ****$p < 0.0001$, two-tailed unpaired $t$-test.

**f,** Cell vaibility of NSTCs cultured with different dose of rWISP1 protein for 4 days. n=5 biological independent samples. Data are shown as means ± s.d.

Source data are provided as a Source Data file.

**Supplementary Figure 3**

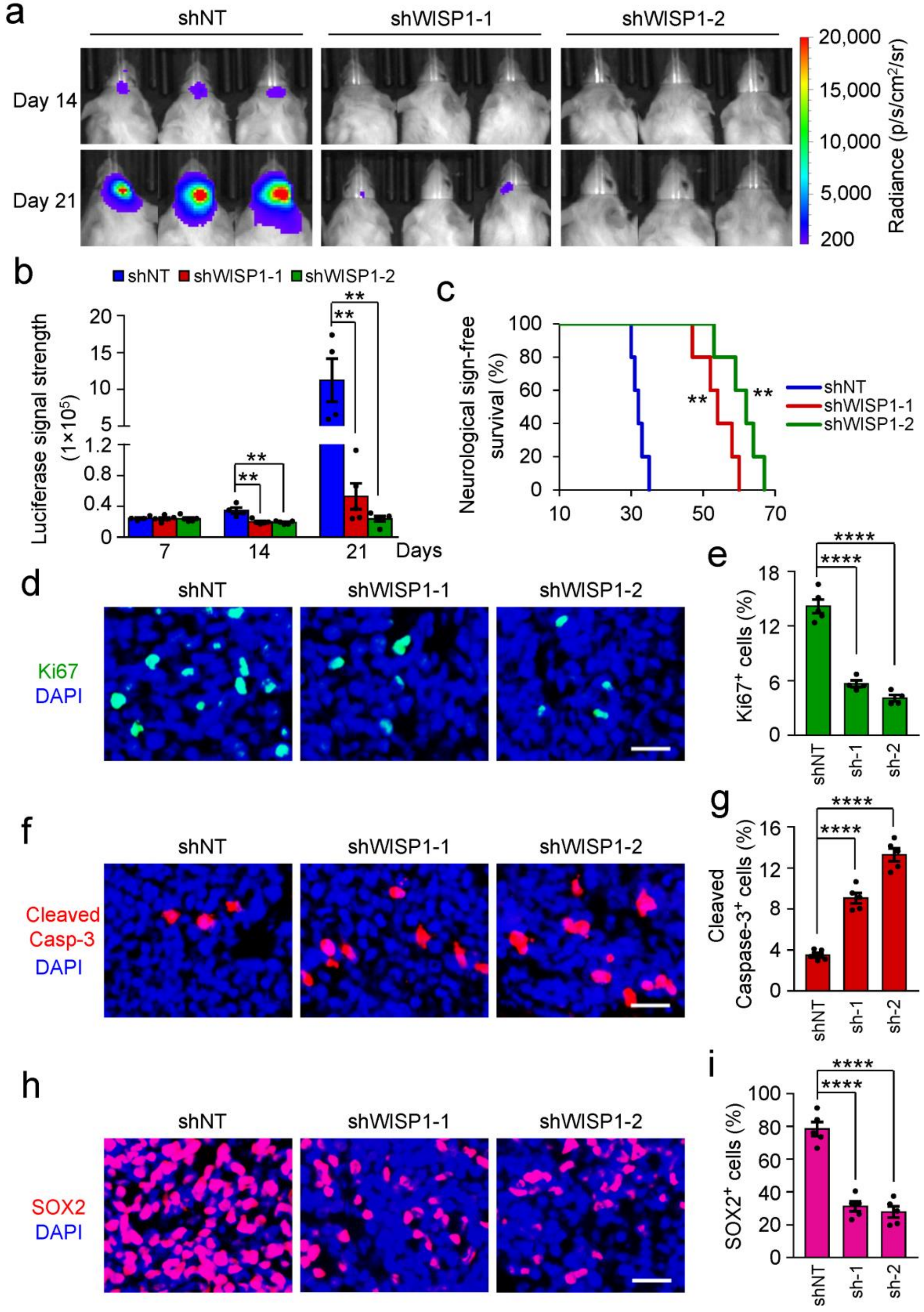

**Supplementary Figure 3. Targeting WISP1 inhibited GSC-driven tumor growth.**

**a, b,** In vivo bioluminescent images (**a**) and quantification (**b**) of the xenografts derived from luciferase-labeled T387 GSCs expressing shNT control or shWISP1 at indicated days after implantation. n=4 (shNT) or 5 (shWISP1-1 or shWISP1-2) mice. Data are represented as means ± s.e.m. shWISP1-1 versus shNT: $^{**}p=0.0051$ (Day 14), $^{**}p=0.0042$ (Day 21), two-tailed unpaired *t*-test. shWISP1-2 versus shNT: $^{**}p=0.0029$ (Day 14), $^{**}p=0.0036$ (Day 21), two-tailed unpaired *t*-test. p, photons; sr, steradian.

**c,** Kaplan-Meier survival curves of mice implanted with T387 GSCs expressing shNT or shWISP1. n=5 mice. shWISP1 group vs. shNT control group, $^{**}p=0.0018$, log-rank test.

**d, e,** Immunofluorescent staining of Ki67 (Green, **d**) and quantification of $Ki67^{+}$ cells (**e**) in GBM tumors derived from T387 GSCs expressing shNT or shWISP1. n=5 (shNT) or 4 (shWISP1-1 or shWISP1-2) biological independent tumor tissues. Scale bar: 25 μm. Data are shown as means ± s.e.m. $^{****}p<0.0001$, two-tailed unpaired *t*-test.

**f, g,** Immunofluorescent staining of Cleaved Caspase-3 (Red, **f**) and quantification of the Cleaved $Caspase\text{-}3^{+}$ cells (**g**) in GBM tumors derived from T387 GSCs expressing shNT or shWISP1. n=5 biological independent tumor tissues. Scale bar: 25 μm. Data are represented as means ± s.e.m. $^{****}p<0.0001$, two-tailed unpaired *t*-test.

**h, i,** Immunofluorescent staining of SOX2 (Red, **h**) and quantification of $SOX2^{+}$ cells (**i**) in GBM tumors derived from T387 GSCs expressing shNT or shWISP1. n=5 biological independent tumor tissues. Scale bar: 25 μm. Data are represented as means ± s.e.m. $^{****}p<0.0001$, two-tailed unpaired *t*-test.

Source data are provided as a Source Data file.

## Supplementary Figure 4

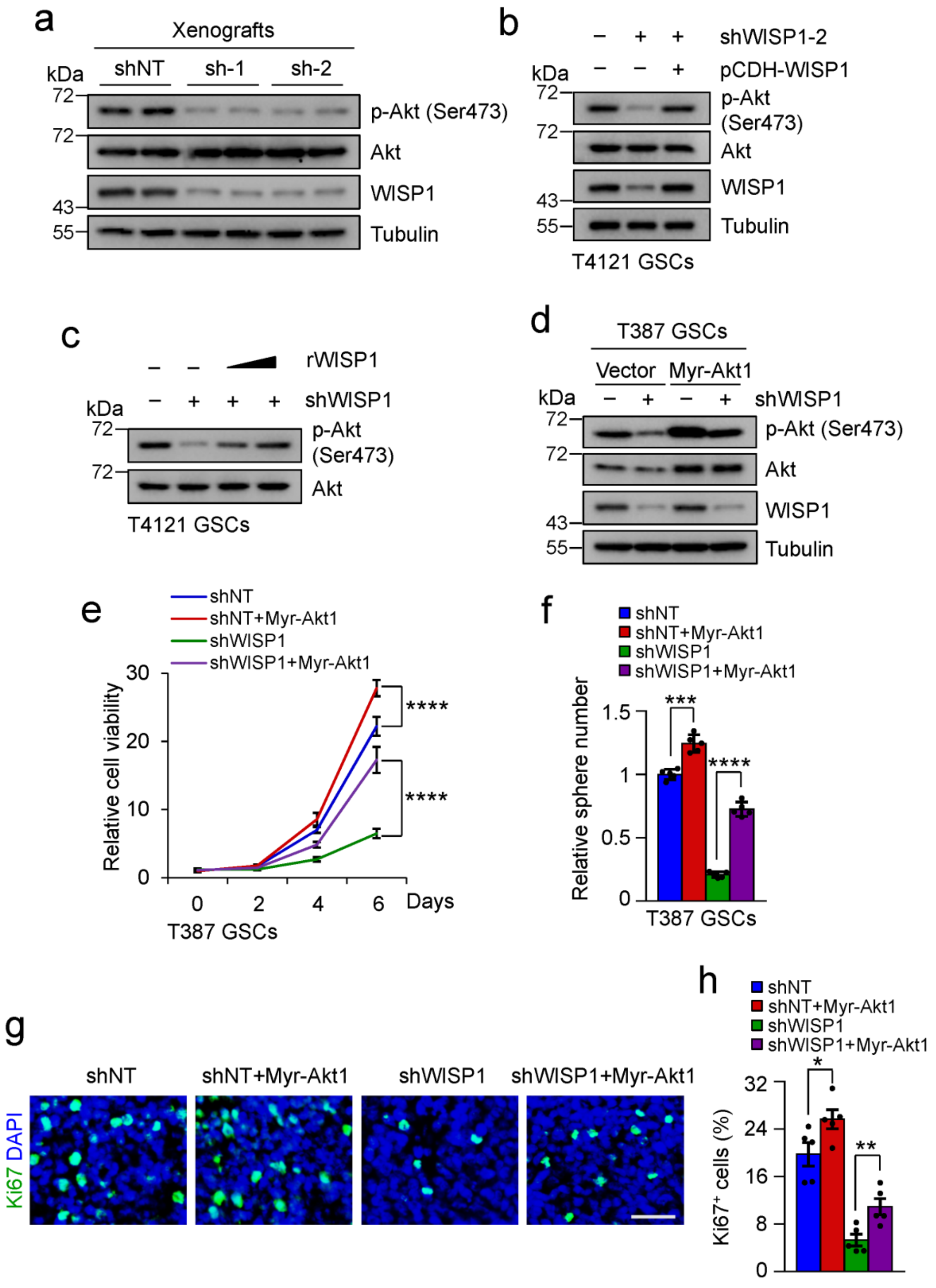

**Supplementary Figure 4. WISP1 activates Akt pathway in GSCs.**

**a,** Immunoblot analysis of Akt activating phosphorylation (p-Akt-Ser473) and WISP1 levels in xenografts derived from T4121 GSCs expressing shNT or shWISP1.

**b,** Immunoblot analysis of Akt phosphorylation (Ser473) in T4121 GSCs transduced with shWISP1-2 or shNT and then transduced with WISP1 overexpression or vector control. GSCs were transduced with shWISP1-2 or shNT lentivirus for 36 hours and then transduced with WISP1 overexpression or control lentivirus for additional 36 hours.

**c,** Immunoblot analysis of Akt phosphorylation (Ser473) in T4121 GSCs transduced with shNT or shWISP1 and then cultured with different dose of rWISP1 (200 ng/ml or 800 ng/ml) protein. GSCs were transduced with shNT or shWISP1 lentivirus for 36 hours and then cultured in Neurobasal media without supplements for 12 hours. The next day, GSCs were treated with rWISP1 (400 ng/ml) for additional 6 hours.

**d,** Immunoblot analysis of Akt activating phosphorylation and WISP1 expression in T387 GSCs transduced with vector control or Myr-Akt1 in combination with shNT or shWIPS1.

**e, f,** T387 GSCs were treated as described in (**d**), and cell viability was detected by cell titler assay (**e**). Relative tumorsphere number is shown (**f**). n=6 biological independent samples. Data are shown as means ± s.d. ****$p < 0.0001$, two way ANOVA analysis followed by Tukey's multiple test (**e**). ***$p = 0.0001$, ****$p < 0.0001$, two-tailed unpaired *t*-test (**f**).

**g, h,** Immunofluorescent staining of Ki67 (Green, **g**) and the quantification of Ki67$^{+}$ cells (**h**) in GBM tumors derived from T4121 GSCs transduced with vector control or Myr-Akt1 in combination with shNT or shWIPS1. n=5 biological independent tumor tissues. Scale bar: 40μm. Data are shown as means ± s.e.m. *$p = 0.0486$, **$p = 0.0098$, two-tailed unpaired *t*-test.

Source data are provided as a Source Data file.

**Supplementary Figure 5**

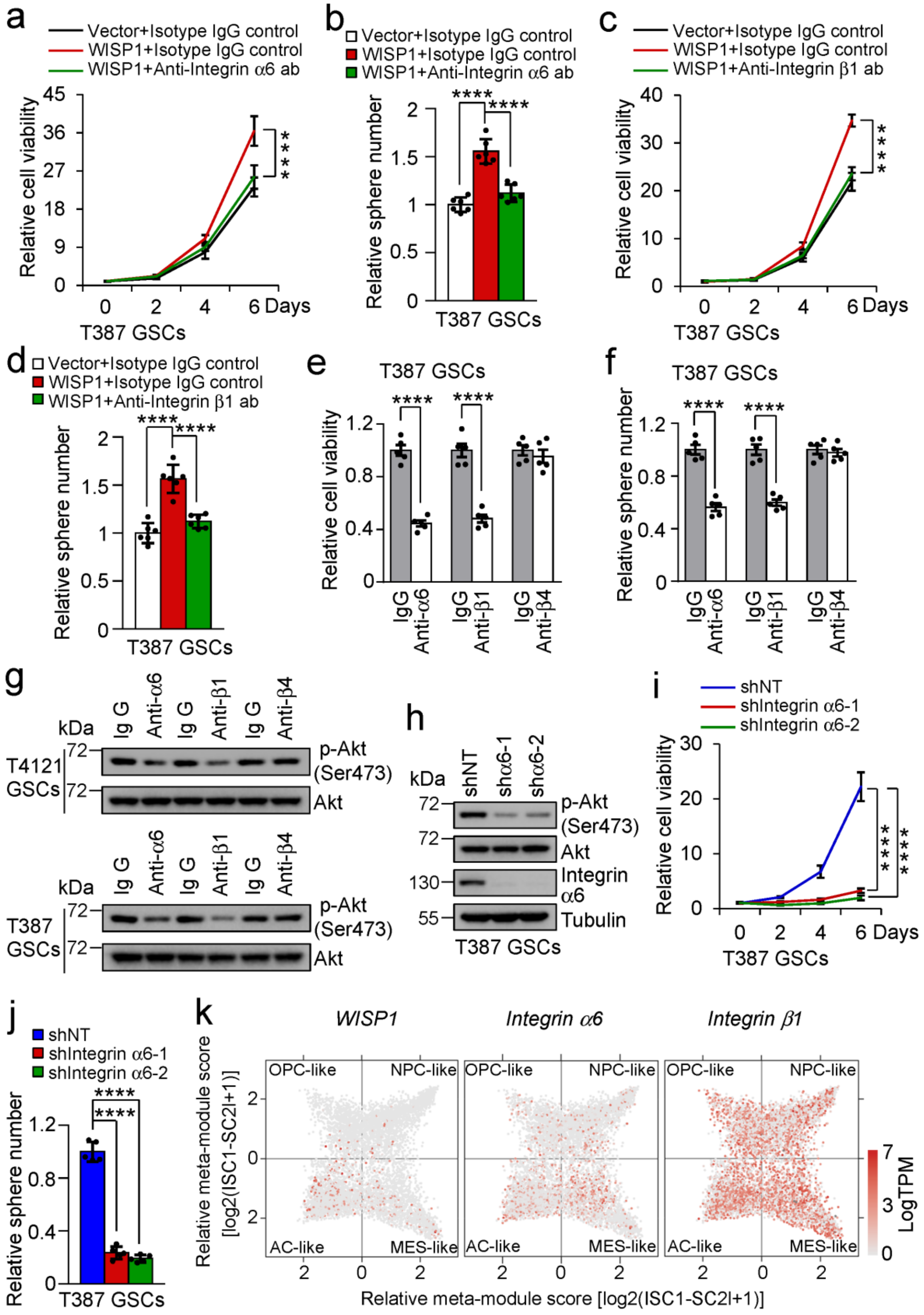

**Supplementary Figure 5. WISP1 activates Akt through Integrin α6β1.**

**a, b,** Cell viability (**a**) or tumorsphere formation (**b**) assay of T387 GSCs treated with 5 μg/ml Integrin α6 blocking antibody (ab) or isotype IgG in combination with WISP1 overexpression or vector control. n=6 biological independent samples. Data are shown as means ± s.d. ****$p < 0.0001$, two way ANOVA analysis followed by Tukey's multiple test (**a**). ****$p < 0.0001$, two-tailed unpaired *t*-test (**b**).

**c, d,** Cell viability (**c**) or tumorsphere formation (**d**) assay of T387 GSCs treated with Integrin β1 blocking antibody (5 μg/ml) or isotype IgG in combination with WISP1 overexpression or vector control. n=5 (**c**) or 6 (**d**) biological independent samples. Data are represented as means ± s.d. ****$p < 0.0001$, two way ANOVA analysis followed by Tukey's multiple test (**c**). ****$p < 0.0001$, two-tailed unpaired *t*-test (**d**).

**e, f,** Cell viability (**e**) or tumorsphere formation (**f**) assay of T387 GSCs treated with Integrin blocking antibody (5 μg/ml) or isotype IgG for 6 days. n=5 biological independent samples. Data are represented as mean ± s.d. ****$p < 0.0001$, two-tailed unpaired *t*-test.

**g,** Immunoblot analysis of Akt phosphorylation (Ser473) in GSCs treated with Integrin blocking antibody (5 μg/ml) or isotype IgG control for 12 hours.

**h,** Immunoblot analysis of Akt phosphorylation (Ser473) and Integrin α6 expression in T387 GSCs transduced with shIntegrin α6 or shNT control.

**i, j,** Cell viability (**i**) or tumorsphere formation (**j**) assay of T387 GSCs transduced with shIntegrin α6 or shNT. n=5 biological independent samples. Data are shown as means ± s.d. ****$p < 0.0001$, two way ANOVA analysis followed by Tukey's multiple test (**i**). ****$p < 0.0001$, two-tailed unpaired *t*-test (**j**).

**k,** The expression of *WISP1*, *Intergrin α6* and *β1* in cluster of two-dimensional representation of cellular states. Each quadrant corresponds to one cellular state, the exact position of malignant cells (dots) reflect their relative scores for the meta-modules, and their colors reflect the gene expression levels. TPM, transcripts per million.

Source data are provided as a Source Data file.

**Supplementary Figure 6**

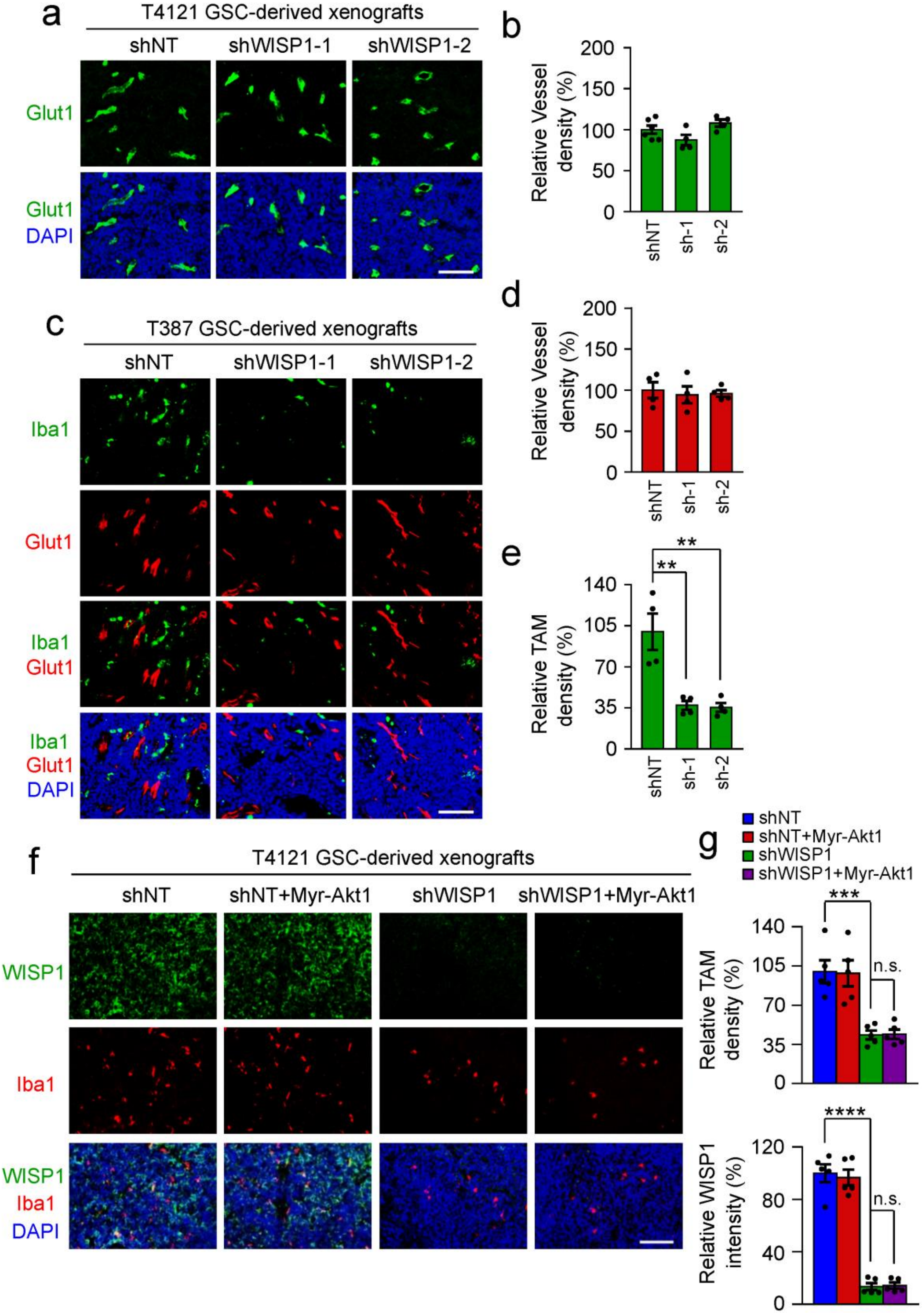

**Supplementary Figure 6. Disrupting WISP1 did not affect vessel density, and constitutively active Akt1 did not impact TAM density.**

**a,** Immunofluorescent staining of vessel marker Glut1 (green) in GBM xenografts derived from T4121 GSCs expressing shNT control or shWISP1. Blood vessels were not affected by WISP1 disruption. Scale Bar, 80 μM.

**b,** Quantitation of Glut1$^{+}$ vessel density in xenografts derived from T4121 GSCs expressing shNT or shWISP1. n=6 (shNT) or 4 (shWISP1-1 or shWISP1-2) biological independent tumor tissues. Data are shown as means ± s.e.m.

**c,** Immunofluorescent staining of the TAM marker Iba1 (green) and the vessel marker Glut1 (red) in GBM xenografts derived from T387 GSCs expressing shNT control or shWISP1. Scale Bar, 80 μM.

**d, e,** Quantitation of Glut1$^{+}$ vessel density (**d**) or Iba1$^{+}$ TAM density (**e**) in xenografts derived from T387 GSCs expressing shNT or shWISP1. n=4 biological independent tumor tissues. Data are shown as means ± s.e.m. ***p*=0.0076 (shWISP1-1 versus shNT), ***p*=0.0066 (shWISP1-2 versus shNT), two-tailed unpaired *t*-test.

**f,** Immunofluorescent staining of WISP1 (green) and the TAM marker Iba1 (red) in GBM xenografts derived from T4121 GSCs transduced with vector control or Myr-Akt1 in combination with shNT or shWISP1. Scale Bar, 100 μM.

**g,** Quantitation of Iba1$^{+}$ TAM density or WISP1 intensity in xenografts derived from T4121 GSCs transduced with vector control or Myr-Akt1 in combination with shNT or shWIPS1. n=5 biological independent tumor tissues. Data are shown as means ± s.e.m. ***$p$=0.0009, ****$p<$0.0001, two-tailed unpaired *t*-test. n.s., not significant.

Source data are provided as a Source Data file.

**Supplementary Figure 7**

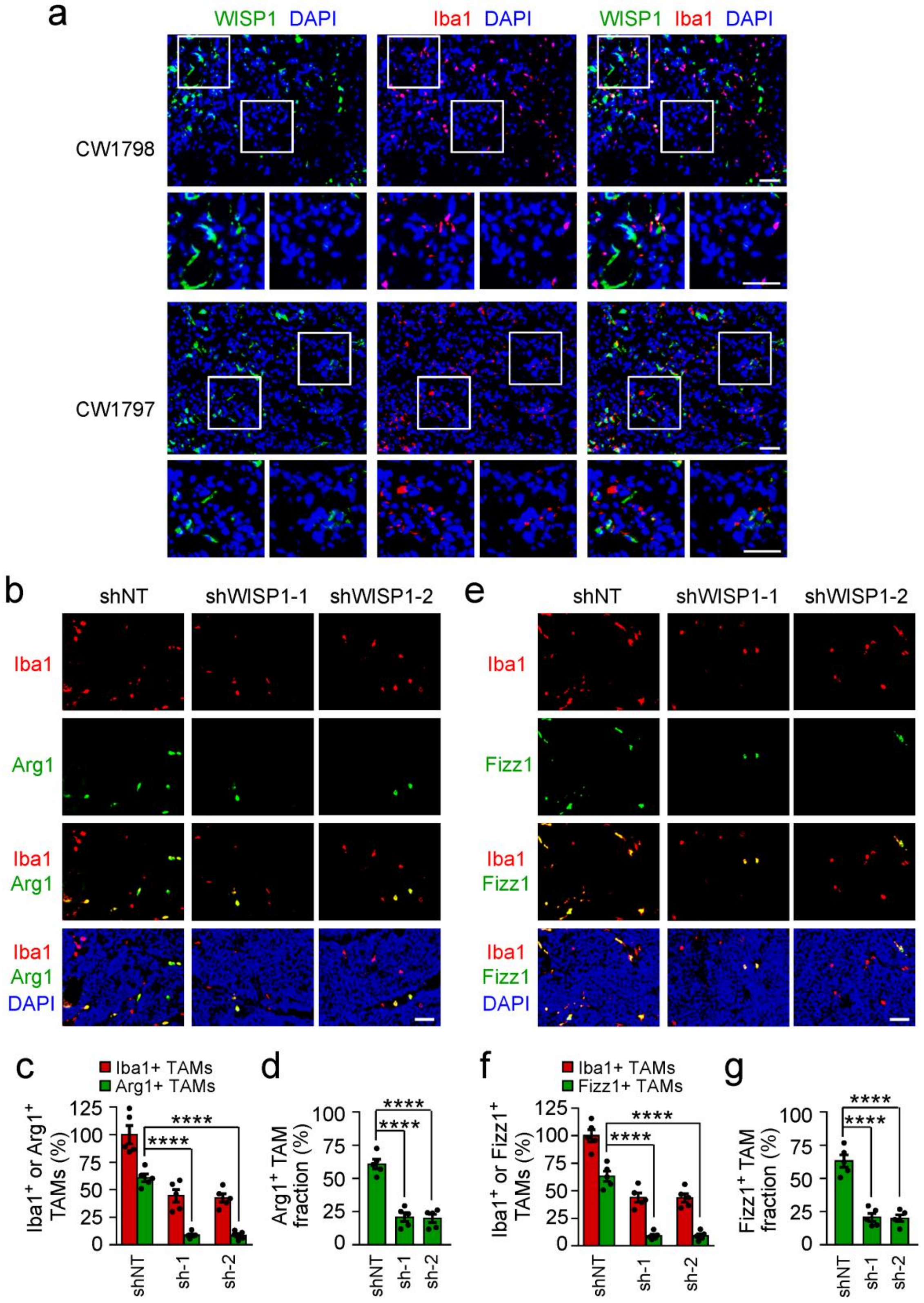

**Supplementary Figure 7. TAMs are enriched in WISP1-abundant regions in GBMs, and silencing WISP1 reduces M2 TAMs in xenografts.**

**a,** Immunofluorescent staining of WISP1 (green) and the TAM marker Iba1 (red) in human primary GBMs. Areas indicated with squares are enlarged and shown on the bottom of each picture. Scale Bar, 50 μM.

**b,** Immunofluorescent staining of the M2 TAM Marker Arg1 (green) and the pan-macrophage marker Iba1 (red) in GBM xenografts derived from T4121 GSCs expressing shNT control or shWISP1. Xenografts were collected from mice when neurological signs occur after GSC transplantation. Scale Bar, 50 μM.

**c, d,** Quantitation of $Arg1^+$ TAM density (**c**) and the fraction of $Arg1^+$ TAMs (**d**) in xenografts derived from T4121 GSCs expressing shNT or shWISP1. n=5 biological independent tumor tissues. Data are represented as means ± s.e.m. ****$p < 0.0001$, two-tailed unpaired *t*-test.

**e,** Immunofluorescent staining of the M2 TAM Marker Fizz1 (green) and the pan-macrophage marker Iba1 (red) in xenografts derived from T4121 GSCs expressing shNT or shWISP1. Xenografts were collected from mice when neurological signs occur after GSC transplantation. Scale Bar, 50 μM.

**f, g,** Quantitation of $Fizz1^+$ TAM density (**f**) and the fraction of $Fizz1^+$ TAMs (**g**) in xenografts derived from T4121 GSCs expressing shNT or shWISP1. n=5 biological independent tumor tissues. Data are represented as means ± s.e.m. ****$p < 0.0001$, two-tailed unpaired *t*-test.

Source data are provided as a Source Data file.

**Supplementary Figure 8**

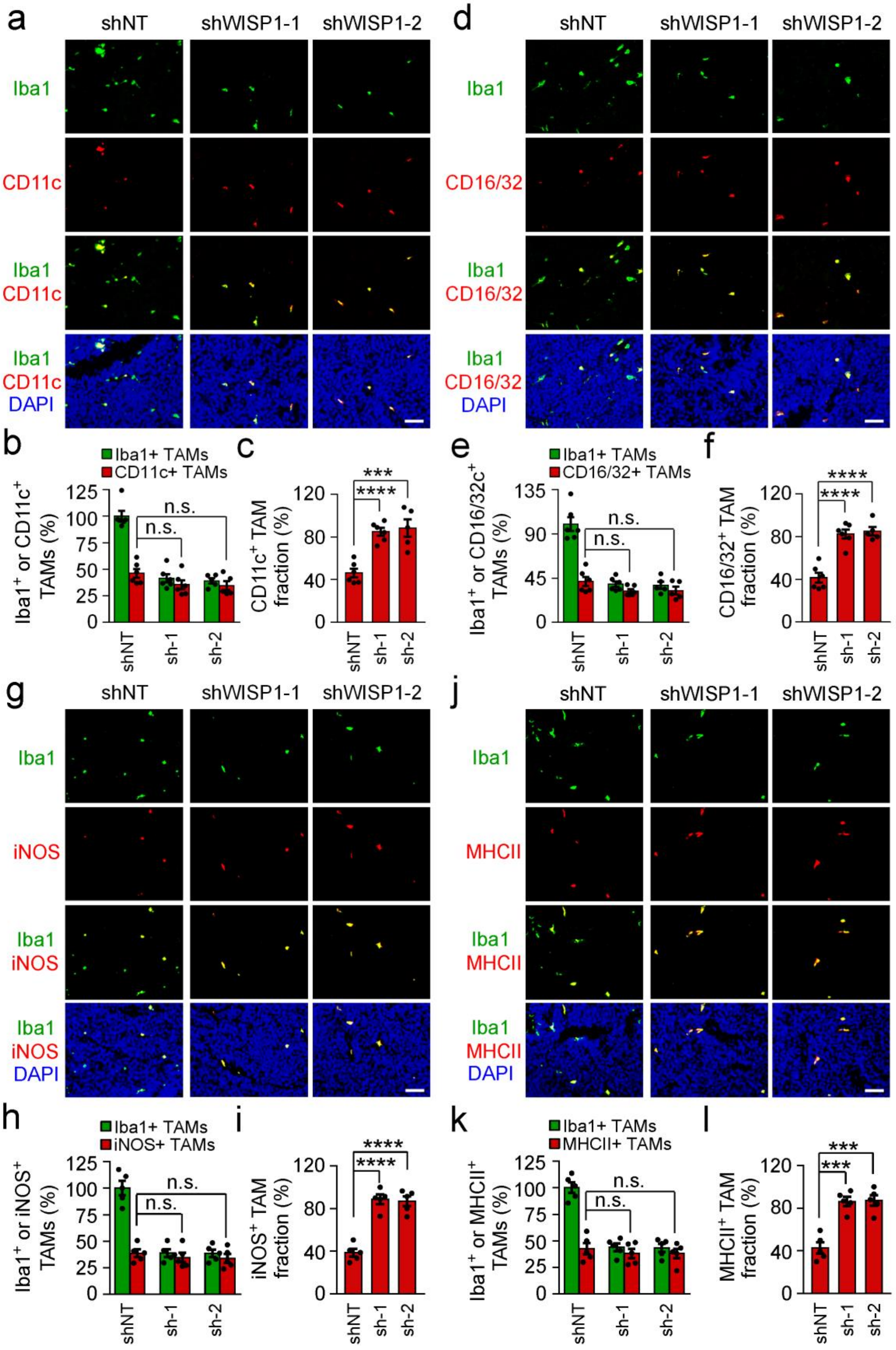

**Supplementary Figure 8. Silencing WISP1 did not affect M1 TAMs in GSC-derived xenografts.**

**a,** Immunofluorescent staining of the M1 TAM Marker CD11c (red) and pan-macrophage marker Iba1 (green) in GBM xenografts derived from T4121 GSCs expressing shNT or shWISP1. Scale Bar, 50 μM.

**b, c,** Quantitation of $CD11c^{+}$ or $Iba1^{+}$ TAM density (**b**) and relative $CD11c^{+}$ TAM fraction (**c**) in xenografts derived from T4121 GSCs expressing shNT or shWISP1. n=6 biological independent tumor tissues. The M1 TAM fraction was determined by the percentage of M1 TAMs within the TAM population in shNT or shWISP1 xenografts, respectively. Data are represented as means ± s.e.m. ****p*=0.0009, ****$p < 0.0001$, two-tailed unpaired *t*-test.

**d,** Immunofluorescent staining of the M1 TAM Marker CD16/32 (red) and pan-macrophage marker Iba1 (green) in GBM xenografts derived from T4121 GSCs expressing shNT or shWISP1. Scale Bar, 50 μM.

**e, f,** Quantitation of $CD16/32^{+}$ or $Iba1^{+}$ TAM density (**e**) and relative $CD16/32^{+}$ TAM fraction (**f**) in xenografts derived from T4121 GSCs expressing shNT or shWISP1. n=6 (shNT or shWISP1-1) or 5 (shWISP1-2) biological independent tumor tissues. Data are represented as means ± s.e.m. ****$p < 0.0001$, two-tailed unpaired *t*-test.

**g,** Immunofluorescent staining of M1 TAM Marker iNOS (red) and pan-macrophage marker Iba1 (green) in GBM xenografts derived from T4121 GSCs expressing shNT or shWISP1. Scale Bar, 50 μM.

**h, i,** Quantitation of $iNOS^{+}$ or $Iba1^{+}$ TAM density (**h**) and relative $iNOS^{+}$ TAM fraction (**i**) in xenografts derived from T4121 GSCs expressing shNT or shWISP1. n=5 biological independent tumor tissues. Data are represented as means ± s.e.m. ****$p < 0.0001$, two-tailed unpaired *t*-test.

**j,** Immunofluorescent staining of the M1 TAM Marker MHCII (red) and pan-macrophage marker Iba1 (green) in xenografts derived from T4121 GSCs expressing shNT or shWISP1. Scale Bar, 50 μM.

**k, l,** Quantitation of $MHCII^{+}$ or $Iba1^{+}$ TAM density (**k**) and relative $MHCII^{+}$ TAM fraction (**l**) in xenografts derived from T4121 GSCs expressing shNT or shWISP1. n=5 biological independent tumor tissues. Data are represented as means ± s.e.m. ****p*=0.0002 (shWISP1-1 versus shNT), ****p*=0.0003 (shWISP1-2 versus shNT), two-tailed unpaired *t*-test.

Source data are provided as a Source Data file.

**Supplementary Figure 9**

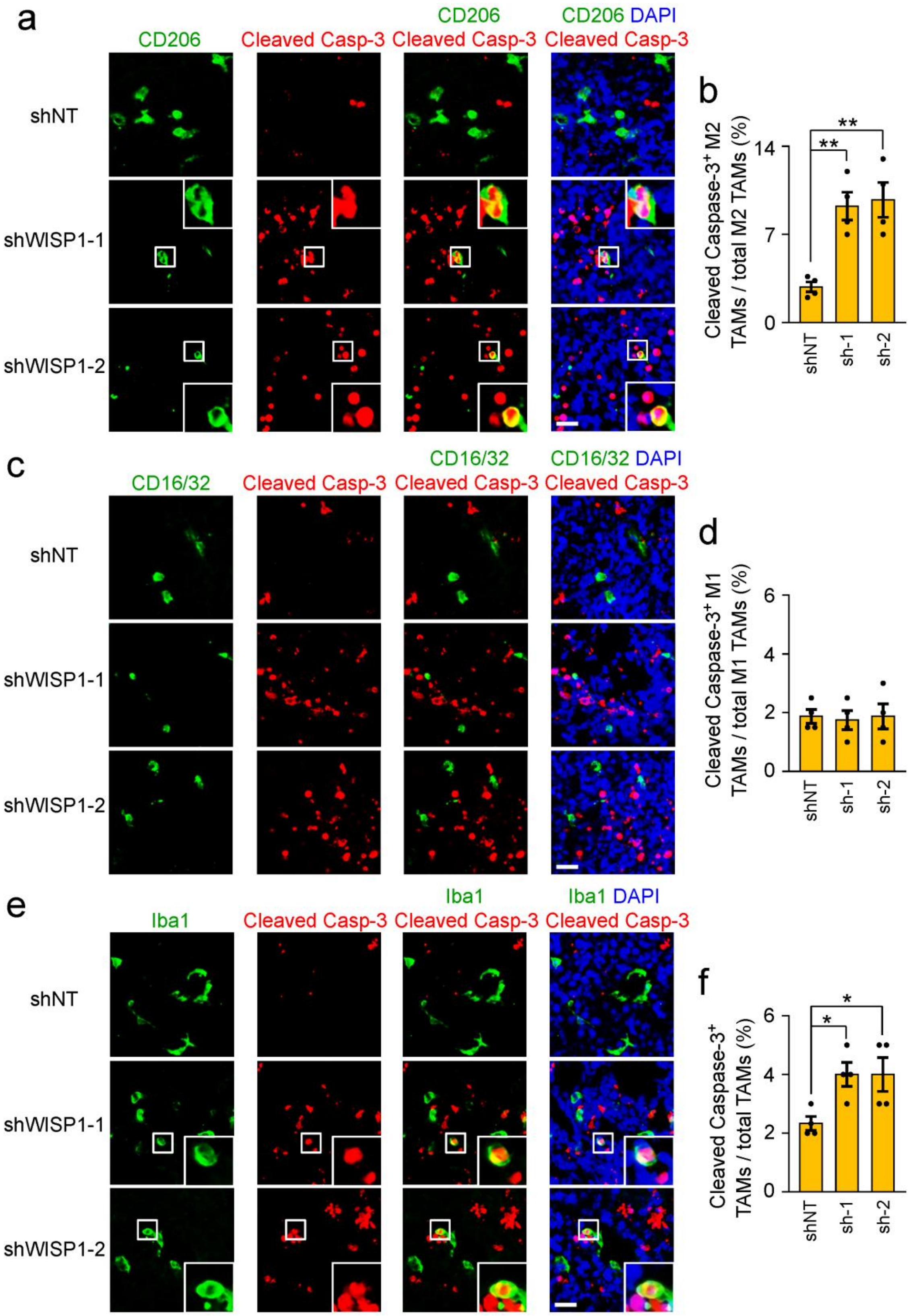

**Supplementary Figure 9. Disrupting WISP1 increased apoptosis of M2 TAMs and total TAMs.**

**a,** Immunofluorescent staining of the M2 TAM marker CD206 (green) and apoptotic marker Cleaved Caspase-3 (red) in tumor xenografts derived from T4121 GSCs expressing shNT or shWISP1. Areas indicated with squares are enlarged and shown on the corner of each picture. Scale Bar, 25 μM.

**b,** Quantification of the apoptotic M2 TAMs (Cleaved Caspase-3$^+$/CD206$^+$) in xenografts derived from T4121 GSCs expressing shNT or shWISP1. n=4 biological independent tumor tissues. Data are represented as means ± s.e.m. **$p$=0.0016 (shWISP1-1 versus shNT), **$p$=0.0029 (shWISP1-2 versus shNT), two-tailed unpaired $t$-test.

**c,** Immunofluorescent staining of the M1 TAM marker CD16/32 (green) and the apoptotic marker Cleaved Caspase-3 (red) in tumor xenografts derived from T4121 GSCs expressing shNT or shWISP1. Scale Bar, 25 μM.

**d,** Quantification of the apoptotic M1 TAMs (Cleaved Caspase-3$^+$/CD16/32$^+$) in xenografts derived from T4121 GSCs expressing shNT or shWISP1. n=4 biological independent tumor tissues. Data are represented as means ± s.e.m.

**e,** Immunofluorescent staining of pan-macrophage marker Iba1 (green) and the apoptotic marker Cleaved Caspase-3 (red) in tumor xenografts derived from T4121 GSCs expressing shNT or shWISP1. Scale Bar, 25 μM.

**f,** Quantification of the total apoptotic TAMs (Cleaved Caspase-3$^+$/Iba1$^+$) in xenografts derived from T4121 GSCs expressing shNT or shWISP1. n=4 biological independent tumor tissues. Data are represented as means ± s.e.m. *$p$=0.0123 (shWISP1-1 versus shNT), *$p$=0.0369 (shWISP1-2 versus shNT), two-tailed unpaired $t$-test.

Source data are provided as a Source Data file.

**Supplementary Figure 10**

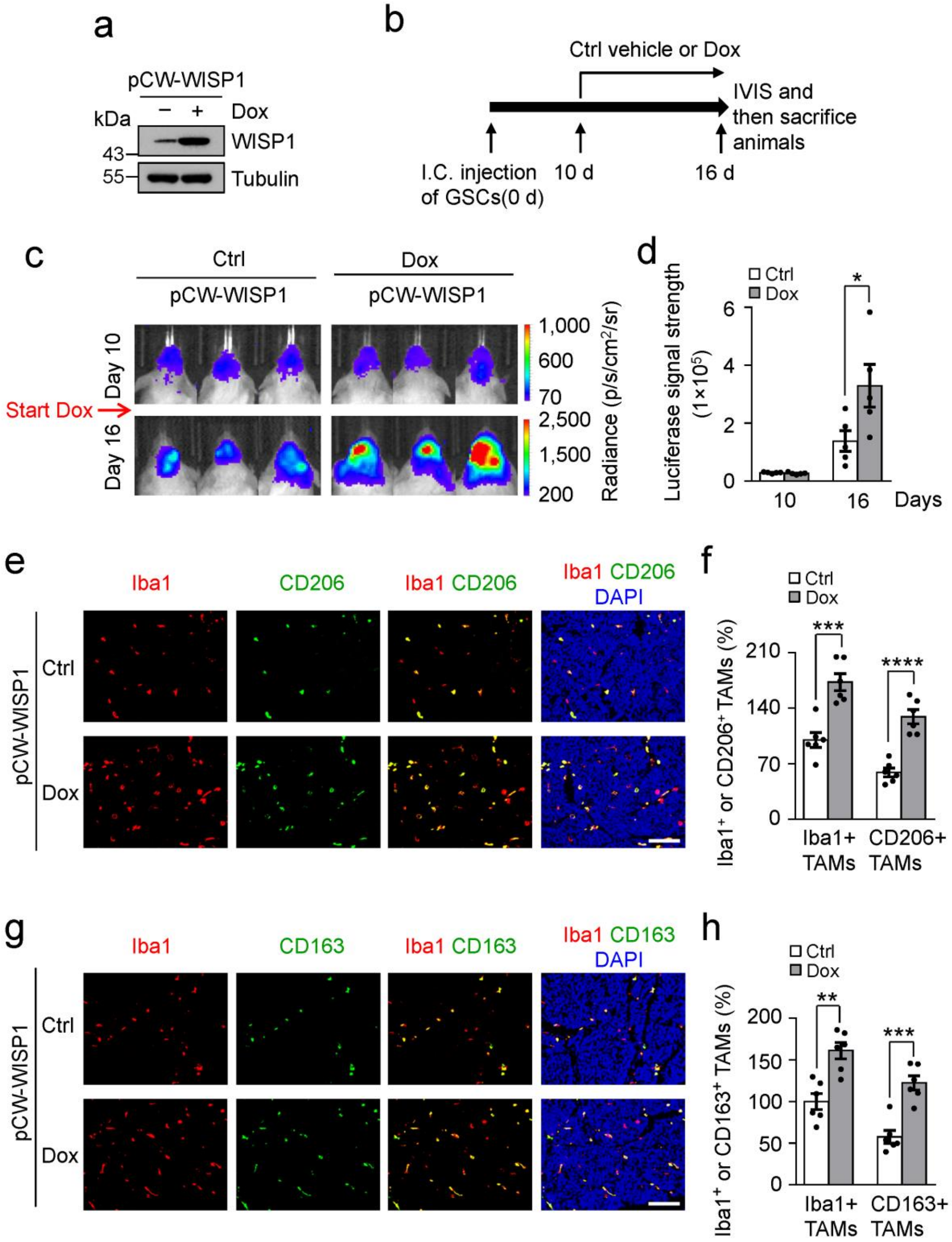

**Supplementary Figure 10. Inducible overexpression of WISP1 increased M2 TAM density and promoted GBM tumor growth.**

**a,** Immunoblot analysis of WISP1 expression in T387 GSC transduced with inducible WISP1 (pCW-WISP1) overexpression and then treated with doxycycline (Dox) for 2 days.

**b,** A schematic diagram showing the treatment of mice bearing luciferase-labeled T387 GSC-derived xenografts with Dox. After GSC implantation for 10 days, mice were then supplied with drinking water with or without doxycycline for 6 days. Tumor growth were monitored through the IVIS bioluminescent imaging system.

**c, d,** In vivo bioluminescent images (**c**) and quantification (**d**) of the T387 GSC-derived xenografts treated with Dox or control vehicle at the indicated days after implantation. n=5 mice. Data are represented as means ± s.e.m. *$p$=0.0486, two-tailed unpaired *t*-test. p, photons; sr, steradian.

**e,** Immunofluorescent staining of the M2 TAM Marker CD206 (green) and pan-macrophage marker Iba1 (red) in T387 GSC-derived tumors treated with Dox or control vehicle. Scale Bar, 100 μM.

**f,** Quantitation of $CD206^+$ TAM or $Iba1^+$ TAM density in xenografts treated with Dox or control vehicle. n=6 biological independent tumor tissues. Data are shown as means ± s.e.m. ***$p$=0.0004, ****$p < 0.0001$, two-tailed unpaired *t*-test.

**g,** Immunofluorescent staining of the M2 TAM Marker CD163 (green) and pan-macrophage marker Iba1 (red) in T387 GSC-derived tumors treated with Dox or control vehicle. Scale Bar, 100 μM.

**h,** Quantitation of $CD163^+$ TAM or $Iba1^+$ TAM density in xenografts treated with Dox or control vehicle. n=6 biological independent tumor tissues. Data are shown as means ± s.e.m. **$p$=0.0013, ***$p$=0.0002, two-tailed unpaired *t*-test.

Source data are provided as a Source Data file.

**Supplementary Figure 11**

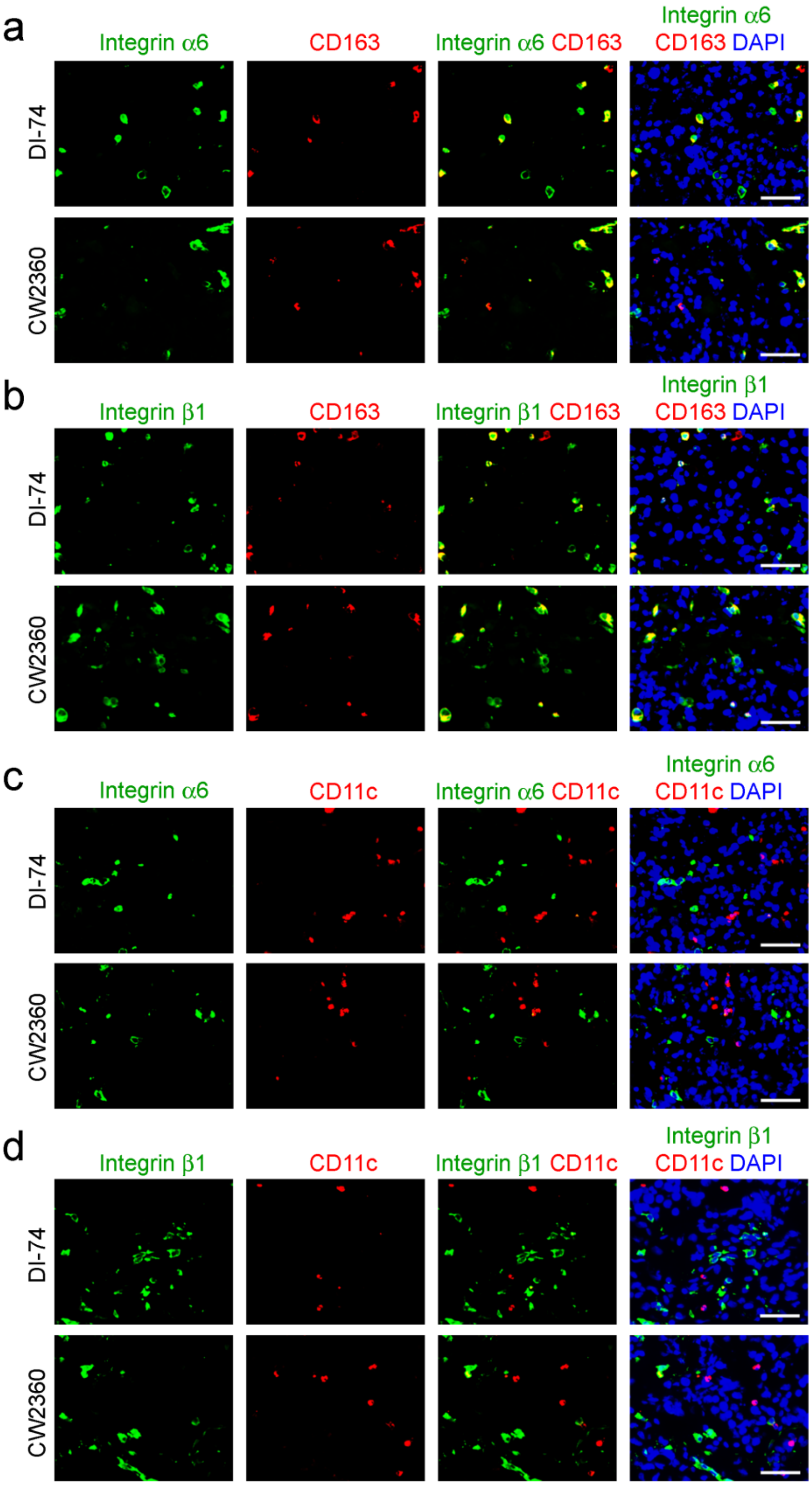

**Supplementary Figure 11. Integrin α6β1 is also expressed on M2 TAMs in human primary GBMs.**

**a,** Immunofluorescent staining of Integrin α6 (green) and the M2 TAM marker CD163 (red) in human primary GBM samples. Scale Bar, 50 μM.

**b,** Immunofluorescent staining of Integrin β1 (green) and the M2 TAM marker CD163 (red) in human primary GBM samples. Scale Bar, 50 μM.

**c,** Immunofluorescent staining of Integrin α6 (green) and the M1 TAM marker CD11c (red) in human primary GBM samples. Scale Bar, 50 μM.

**d,** Immunofluorescent staining of Integrin β1 (green) and the M1 TAM marker CD11c (red) in human primary GBM samples. Scale Bar, 50 μM.

## Supplementary Figure 12

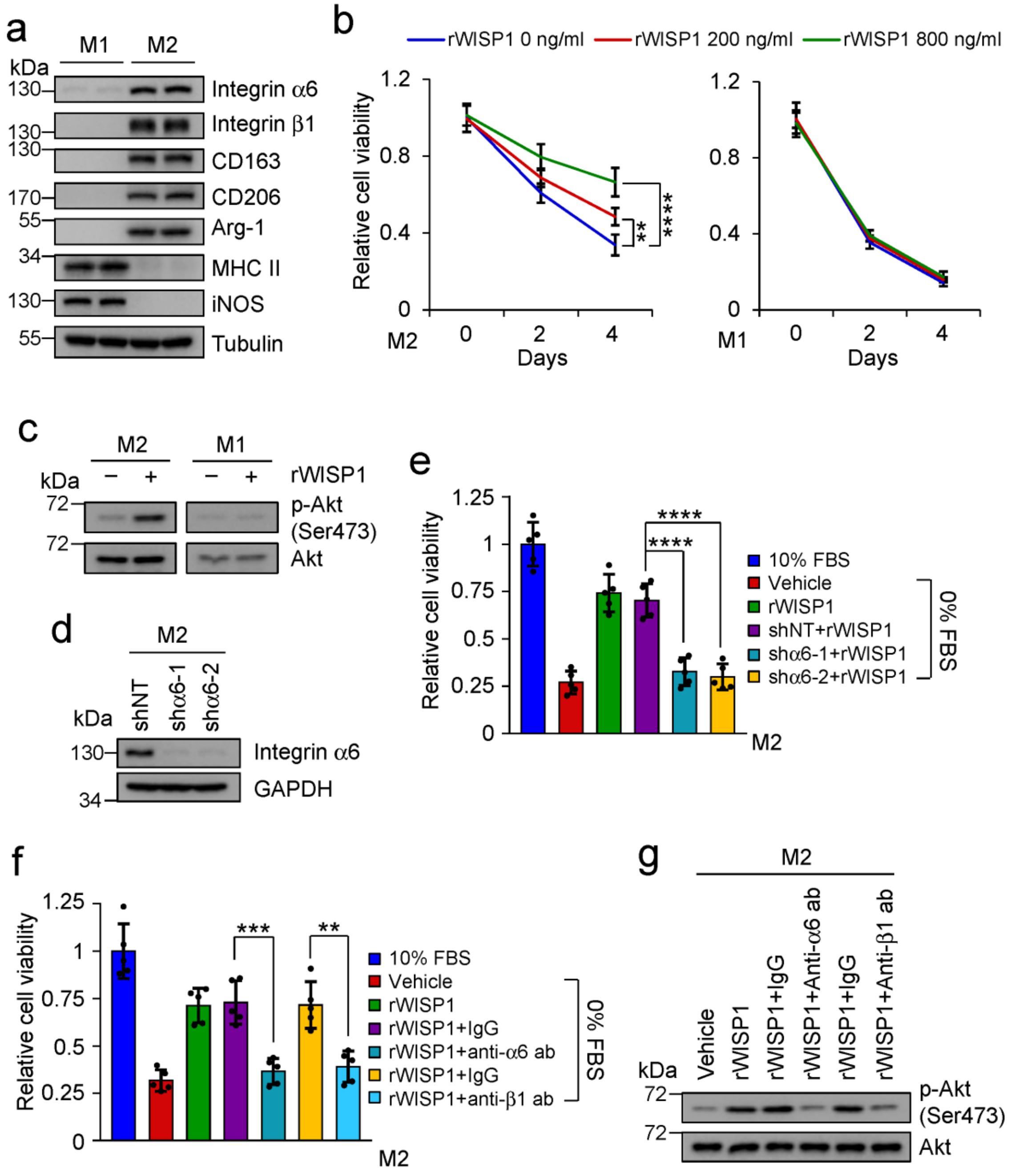


**Supplementary Figure 12. WISP1 promoted M2 macrophage survival through Integrin α6β1-Akt signaling.**

**a,** Immunoblot analysis of Integrin α6, β1, the M2 macrophage marker and M1 macrophage marker expression in U937-derived M1 (M1) or M2 macrophages (M2).

**b,** Cell viability assay of U937-derived M2 macrophages cultured in serum-free media

with different dose of rWISP1 protein for indicated days. n=5 biological independent samples (M2 or M1). Data are shown as means ± s.d. ***p*=0.0053, *****p* < 0.0001, two way ANOVA analysis followed by Tukey's multiple test.

**c,** Immunoblot analysis of Akt activating phosphorylation (p-Akt-Ser473) in U937-derived M1 or M2 macrophages treated with rWISP1 protein. Cells were starved for serum overnight and then treated with rWISP1 (400 ng/ml) for 6 hours.

**d,** Immunoblot analysis of Integrin α6 expression in U937-derived M2 macrophages transduced with shNT or Integrin α6 shRNA (shα6).

**e,** Cell viability assay of U937-derived M2 macrophages transduced with shNT or shIntegrin α6 and cultured with rWISP1 protein. n=5 biological independent samples. Cells were infected with shNT or shIntegrin α6 lentivirus for 24 hours and then cultured in serum-free media with rWISP1 protein (400 μg/ml) for 4 days. Data are represented as means ± s.d. *****p* < 0.0001, two-tailed unpaired *t*-test.

**f,** Cell viability assay of U937-derived M2 macrophages treated with Integrin blocking antibody or isotype IgG in combination with rWISP1 protein. n=5 biological independent samples. Cells were cultured in serum-free media and treated with Integrin blocking antibody (5 μg/ml) or isotype IgG in combination with rWISP1 protein (400 μg/ml) for 4 days. Data are shown as means ± s.d. ***p*=0.0012, ****p*=0.0003, two-tailed unpaired *t*-test.

**g,** Immunoblot analysis of Akt activating phosphorylation in U937-derived M2 macrophages treated with the Integrin blocking antibody or isotype IgG in combination with rWISP1 protein. Cells were starved for serum overnight and then treated with Integrin blocking antibody or IgG for 1 hour follewed by stimulation with rWISP1 (400 μg/ml) for 6 hours.

Source data are provided as a Source Data file.

**Supplementary Figure 13**

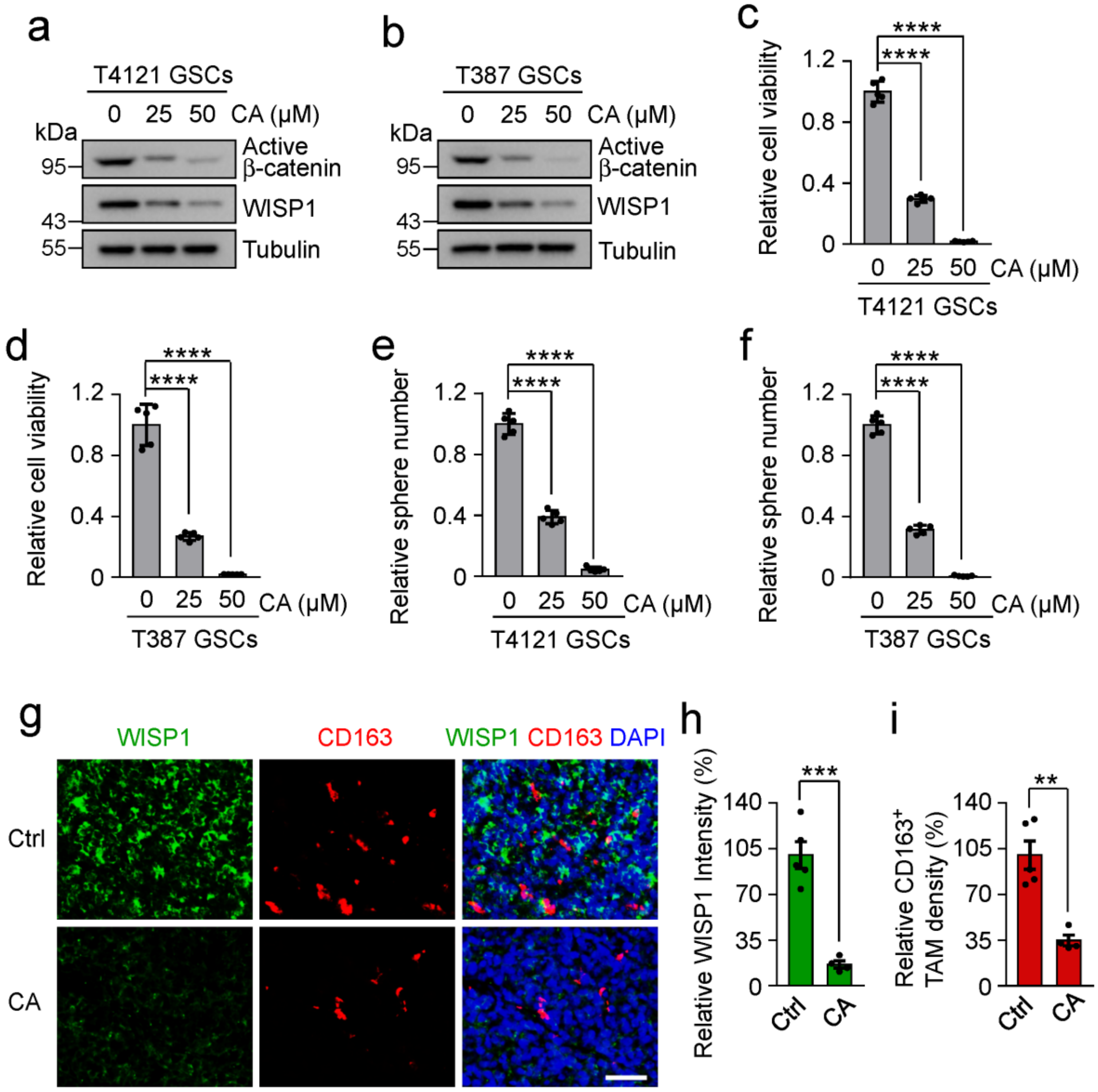


**Supplementary Figure 13. Carnosic acid (CA) disrupted the maintenance of GSCs in vitro and M2 TAMs in vivo.**

**a, b,** Immunoblot analysis of the active β-catenin and WISP1 levels in T4121 GSCs (**a**) or T387 GSCs (**b**) treated with indicated doses of CA or the vehicle control for 12 hours.

**c, d,** Cell viability assay of T4121 GSCs (**c**) or T387 GSCs (**d**) treated with indicated doses of CA or the vehicle control for 4 days. n=5 biological independent samples. Data are represented as means ± s.d. ****$p < 0.0001$, two-tailed unpaired *t*-test.

**e, f,** Tumorsphere formation of T4121 GSCs (**e**) or T387 GSCs (**f**) treated with indicated doses of CA or the vehicle control. n=5 biological independent cell cultures. Data are

shown as means ± s.d. ****$p < 0.0001$, two-tailed unpaired *t*-test.

**g,** Immunofluorescent staining of WISP1 (green) and the M2 TAM marker CD163 (red) in T4121 GSC-derived tumors treated CA or vehicle control. Scale Bar, 50 μM.

**h, i,** Quantitation of WISP1 intensity (**h**) or $CD163^{+}$ TAM density (**i**) in xenografts treated CA or vehicle control. n= 5 (Ctrl) or 4 (CA) biological independent tumor tissues. Data are shown as means ± s.e.m. **$p=0.0013$, ***$p=0.0002$, two-tailed unpaired *t*-test.

Source data are provided as a Source Data file.

## Supplementary Figure 14

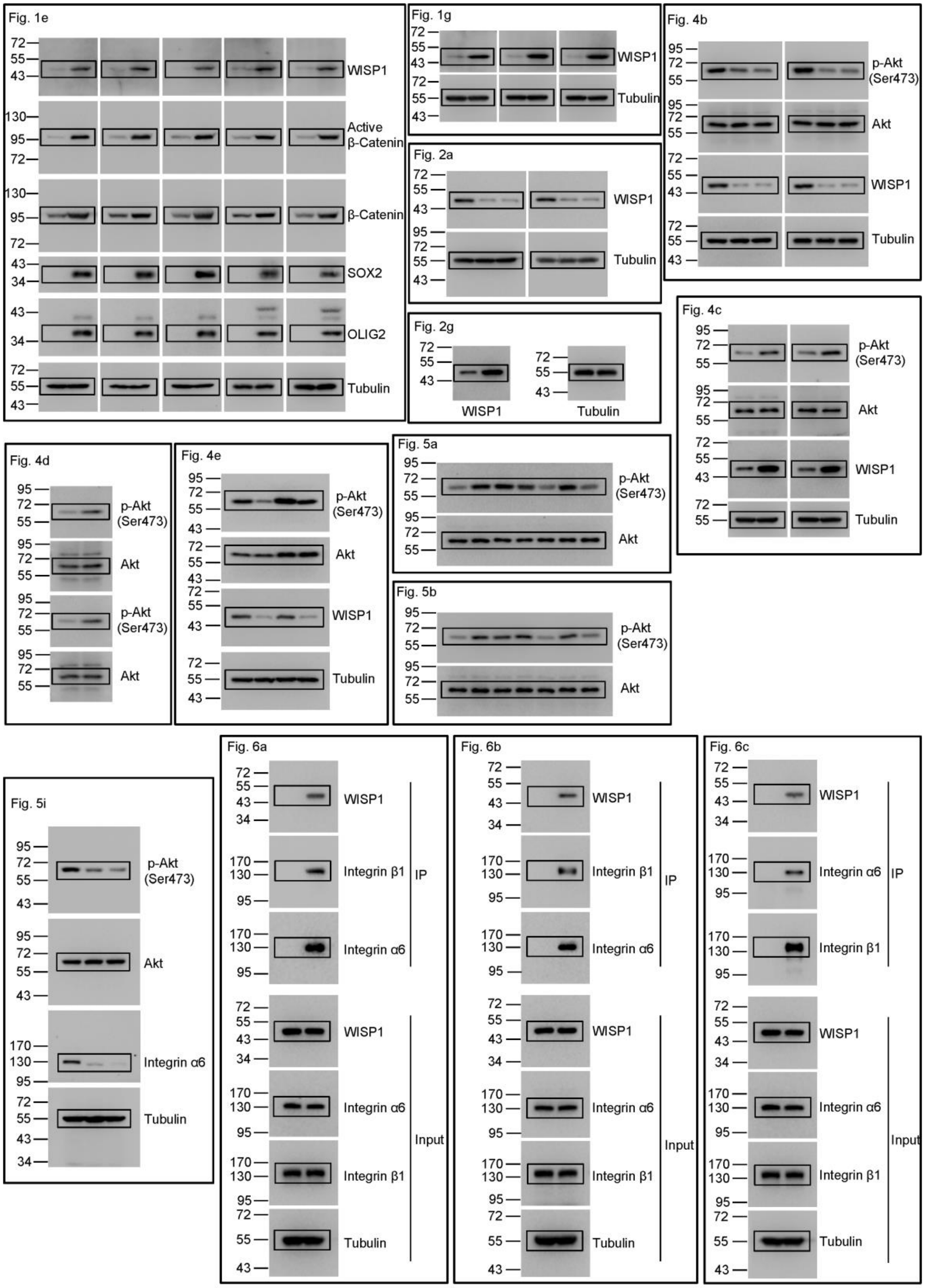

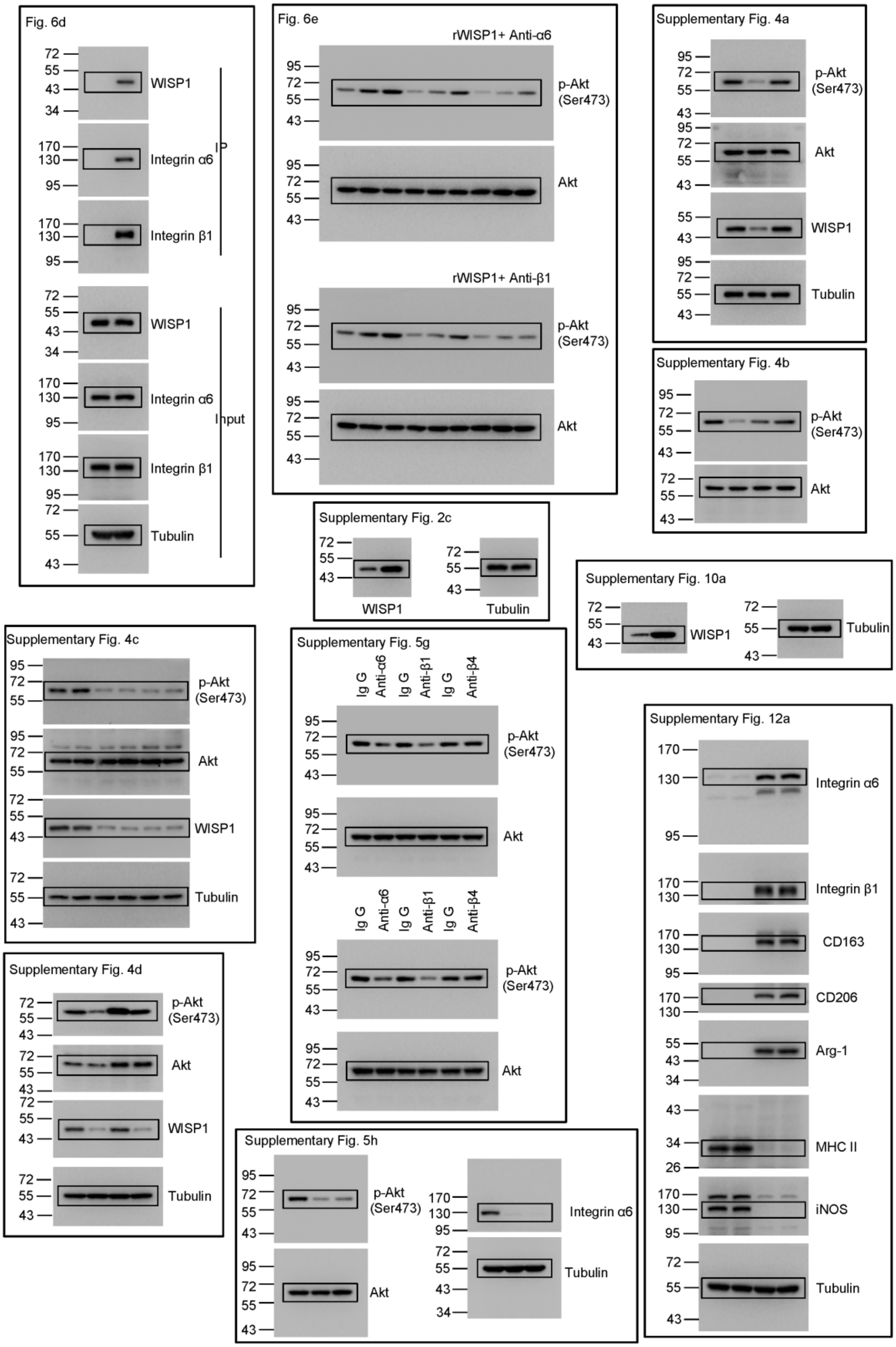

Fig. 6d
72
55
43
34
WISP1
170
130
95
Integrin α6
IP
Integrin β1
Input
Tubulin
Fig. 6e
rWISP1+ Anti-α6
rWISP1+ Anti-β1
p-Akt (Ser473)
Akt
Supplementary Fig. 4a
Supplementary Fig. 4b
Supplementary Fig. 2c
Supplementary Fig. 10a
Supplementary Fig. 4c
Supplementary Fig. 5g
Ig G
Anti-α6
Anti-β1
Anti-β4
Supplementary Fig. 12a
CD163
CD206
Arg-1
MHC II
iNOS
26
Supplementary Fig. 4d
Supplementary Fig. 5h

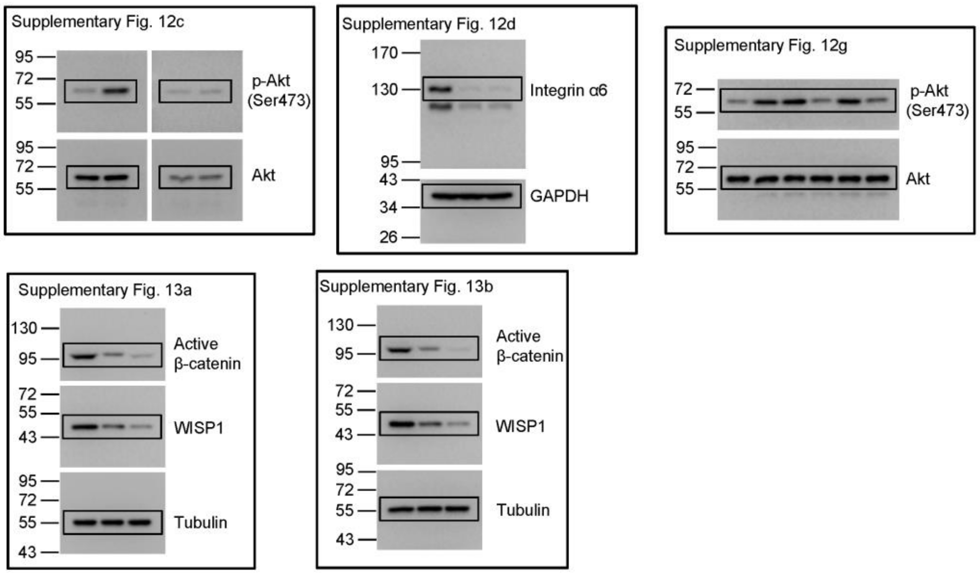


**Supplementary Figure 14. Uncropped images of immunoblots.**

Uncropped images of immunoblots are shown in the order they are presented in the figures.

**Supplementary Table 1. List of all antibodies used in this study.**

| Primary or Secondary | Antibodies | Source | Catalog | Application | Dilution |
|---|---|---|---|---|---|
| 1st | SOX2 | Bethyl | A301-739A | WB | 1:1000 |
| 1st | WISP1 | Santa Cruz | sc-25441 | WB | 1:500 |
| 1st | p-Akt (Ser473) | Cell Signaling | 4058 | WB | 1:1000 |
| 1st | Akt | Cell Signaling | 2966 | WB | 1:1000 |
| 1st | Integrin α6 | Abcam | Ab181551 | WB | 1:1500 |
| 1st | Integrin β1 | Abcam | Ab52971 | WB | 1:1000 |
| 1st | CD206 | Abcam | Ab64693 | WB | 1:500 |
| 1st | CD163 | BIO-RAD | MCA1853 | WB | 1:500 |
| 1st | Arg-1 | BD Biosciences | 610708 | WB | 1:500 |
| 1st | MHC II | Abcam | Ab20181 | WB | 1:500 |
| 1st | iNOS | Abcam | Ab3523 | WB | 1:1000 |
| 1st | Active β-Catenin | Cell Signaling | 4270S | WB | 1:1000 |
| 1st | β-Catenin | Cell Signaling | 9582T | WB | 1:1000 |
| 1st | Tubulin | Sigma-Aldrich | 6199 | WB | 1:3000 |
| 1st | GAPDH | R&D Systems | MAB5718 | WB | 1:3000 |
| 1st | Integrin α6 | Santa Cruz | sc-13542 | IP | N/A |
| 1st | Integrin β1 | Abcam | Ab30388 | IP | N/A |
| 1st | SOX2 | Santa Cruz | sc-17320 | IF | 1:100 |
| 1st | SOX2 | Bethyl | A301-739A | IF | 1:100 |
| 1st | WISP1 | Santa Cruz | sc-25441 | IF | 1:150 |
| 1st | WISP1 | Santa Cruz | sc-133126 | IF | 1:150 |
| 1st | OLIG2 | R&D Systems | AF2418 | IF | 1:100 |
| 1st | Ki67 | Cell Signaling | 9129 | IF | 1:400 |
| 1st | Cleaved Caspase-3 | Cell Signaling | 9661 | IF | 1:200 |
| 1st | Iba1 | Abcam | Ab5076 | IF | 1:200 |
| 1st | Iba1 | Wako Chemicals | 019-19741 | IF | 1:400 |
| 1st | CD11b | BIO-RAD | MCA711GT | IF | 1:100 |
| 1st | CD163 | Thermo fisher | 14-1631-80 | IF | 1:200 |
| 1st | CD163 | Santa Cruz | sc-20066 | IF | 1:75 |
| 1st | CD206 | Santa Cruz | sc-58987 | IF | 1:100 |
| 1st | CD206 | BIO-RAD | MCA2235 | IF | 1:200 |
| 1st | Arg1 | BD Biosciences | 610708 | IF | 1:75 |
| 1st | Fizz1 | Abcam | Ab39626 | IF | 1:100 |
| 1st | CD11c | Thermo fisher | PA5-35326 | IF | 1:100 |
| 1st | CD11c | BD Biosciences | 558079 | IF | 1:100 |
| 1st | CD16/32 | BD Biosciences | 553141 | IF | 1:250 |
| 1st | iNOS | BD Biosciences | 610328 | IF | 1:75 |
| 1st | MHCII | Thermo fisher | 14-5321-82 | IF | 1:100 |
| 1st | GLUT1 | Thermo Fisher | PA1-37782 | IF | 1:500 |

| | | | | | |
|---|---|---|---|---|---|
| 1st | Integrin α3 | Millipore | MAB1952Z | Blocking antibody | N/A |
| 1st | Integrin α6 | Millipore | MAB1378 | Blocking antibody | N/A |
| 1st | Integrin α7 | BIO-RAD | MCA5238Z | Blocking antibody | N/A |
| 1st | Integrin β1 | Millipore | MAB2253 | Blocking antibody | N/A |
| 1st | Integrin β4 | Millipore | MAB2059Z | Blocking antibody | N/A |
| 1st | PE-conjugated anti-CD133 | Miltenyi Biotec | 130-098-826 | Flow cytometry | N/A |
| 1st | FITC-conjugated anti-CD15 | BD Biosciences | 347423 | Flow cytometry | N/A |
| 2nd | Anti-rabbit IgG, HRP-linked Antibody | Cell Signaling | 7074S | WB | 1:3000 |
| 2nd | Anti-mouse IgG, HRP-linked Antibody | Cell Signaling | 7076S | WB | 1:3000 |
| 2nd | Anti-Rat IgG, HRP-linked Antibody | Cell Signaling | 7077S | WB | 1:3000 |
| 2nd | Anti-Goat IgG, HRP-linked Antibody | Santa Cruz | SC-2354 | WB | 1:3000 |
| 2nd | Donkey anti-Mouse IgG (H+L) Secondary Antibody, Alexa Fluor 488 | Thermo fisher | A-21202 | IF | 1:200 |
| 2nd | Donkey anti-Rabbit IgG (H+L) Secondary Antibody, Alexa Fluor 488 | Thermo fisher | A-21206 | IF | 1:200 |
| 2nd | Donkey anti-Goat IgG (H+L) Secondary Antibody, Alexa Fluor 488 | Thermo fisher | A-11055 | IF | 1:200 |
| 2nd | Donkey anti-Rat IgG (H+L) Secondary Antibody, Alexa Fluor 488 | Thermo fisher | A-21208 | IF | 1:200 |
| 2nd | Donkey anti-Mouse IgG (H+L) Secondary Antibody, Alexa Fluor 568 | Thermo fisher | A-10037 | IF | 1:200 |
| 2nd | Donkey anti-Rabbit IgG (H+L) Secondary Antibody, Alexa Fluor 568 | Thermo fisher | A-10042 | IF | 1:200 |
| 2nd | Donkey anti-Goat IgG (H+L) Secondary Antibody, Alexa Fluor 568 | Thermo fisher | A-11057 | IF | 1:200 |
| 2nd | Donkey anti-Rat IgG (H+L) Secondary Antibody, Alexa Fluor 594 | Thermo fisher | A-21209 | IF | 1:200 |
