# Supplementary material for: Dual Role of WISP1 in maintaining glioma stem cells and tumor-supportive macrophages in glioblastoma
Source: Nat Commun. 2020 Jun 15;11:3015. doi: 10.1038/s41467-020-16827-z (PMC7295765; doi:10.1038/s41467-020-16827-z)
Supplement: Supplementary file 3 — Reporting summary [file 41467_2020_16827_MOESM3_ESM.pdf]

nature research

Corresponding author(s): Shideng Bao

Last updated by author(s): May 17, 2020

Reporting Summary

Nature Research wishes to improve the reproducibility of the work that we publish. This form provides structure for consistency and transparency in reporting. For further information on Nature Research policies, see Authors & Referees and the Editorial Policy Checklist .

Statistics

For all statistical analyses, confirm that the following items are present in the figure legend, table legend, main text, or Methods section.

n/a	Confirmed	
☐	☒	The exact sample size (n) for each experimental group/condition, given as a discrete number and unit of measurement
☐	☒	A statement on whether measurements were taken from distinct samples or whether the same sample was measured repeatedly
☐	☒	The statistical test(s) used AND whether they are one- or two-sided<br>*Only common tests should be described solely by name; describe more complex techniques in the Methods section.*
☒	☐	A description of all covariates tested
☐	☒	A description of any assumptions or corrections, such as tests of normality and adjustment for multiple comparisons
☐	☒	A full description of the statistical parameters including central tendency (e.g. means) or other basic estimates (e.g. regression coefficient) AND variation (e.g. standard deviation) or associated estimates of uncertainty (e.g. confidence intervals)
☐	☒	For null hypothesis testing, the test statistic (e.g. F, t, r) with confidence intervals, effect sizes, degrees of freedom and P value noted<br>*Give P values as exact values whenever suitable.*
☒	☐	For Bayesian analysis, information on the choice of priors and Markov chain Monte Carlo settings
☒	☐	For hierarchical and complex designs, identification of the appropriate level for tests and full reporting of outcomes
☒	☐	Estimates of effect sizes (e.g. Cohen's d, Pearson's r), indicating how they were calculated

Our web collection on statistics for biologists contains articles on many of the points above.

Software and code

Policy information about availability of computer code

Data collection	Image J 1.47v was used to quantify the positive cells in immunofluorescent staining assay. BioRad Image Lab 5.2.1 was used for image acquisition of protein Blots.
Data analysis	GraphPad Prism 7.0 or Microsoft Excel 2010 was used to perform statistical analysis in the paper.

For manuscripts utilizing custom algorithms or software that are central to the research but not yet described in published literature, software must be made available to editors/reviewers. We strongly encourage code deposition in a community repository (e.g. GitHub). See the Nature Research guidelines for submitting code & software for further information.

Data

Policy information about availability of data

All manuscripts must include a data availability statement. This statement should provide the following information, where applicable:

- Accession codes, unique identifiers, or web links for publicly available datasets
- A list of figures that have associated raw data
- A description of any restrictions on data availability

The TCGA database (Agilent-4502A platform) and Gravendeel database can be downloaded from GlioVis data portal (http://gliovis.bioinfo.cnio.es/). The gene expression in cluster of two-dimensional representation of cellular states can be downloaded from Single Cell Portal (http://singlecell.broadinstitute.org/single_cell/study/SCP393/single-cell-rna-seq-of-adult-and-pediatric-glioblastoma/). All other data supporting the findings of this study are available within the article and its supplementary information files. All remaining data are available from the corresponding author upon reasonable request. The source data underlying Figs.1a-d, i, 2b, d-f, h, i, 3b, c, e, g, i, 4f, g, i, j, 5c-h, j, k, 7b, c, e, f, h, i, k, l, 8c, d, f, h, j, l, and Supplementary Figs. 1a, b, 2a, b, d-f, 3b, c, e, g, i, 4e, f, h, 5a-f, i, j, 6b, d, e, g, 7c, d, f, g, 8b, c, e, f, h, i, k, l, 9b, d, f, 10d, f, h, 12b, e, f, 13c-f, h, i, are provided as a Source Data file.

Field-specific reporting

Please select the one below that is the best fit for your research. If you are not sure, read the appropriate sections before making your selection.

☒ Life sciences ☐ Behavioural & social sciences ☐ Ecological, evolutionary & environmental sciences

For a reference copy of the document with all sections, see nature.com/documents/nr-reporting-summary-flat.pdf

Life sciences study design

All studies must disclose on these points even when the disclosure is negative.

Sample size	We have utilized Guidelines for the Care and Use of Mammals in Neuroscience and Behavioral Research from the National Research Council to estimate the minimal number of animals necessary to achieve statistical significance. The number of animals per arm is based upon the following calculation: N=1+2C(s/d)^2 where n=number of animals per arm, C=7.85 when alpha=0.05 and 1-beta=0.8 (significance level of 5% with a power of 80%), s= standard deviation, and d=difference to be detected. The precise number of animals used are described in each figure legend. In in vitro experiments, the sample size was chosen based on previous experience with similar experiments, and in order to reproducibly detect specific effects. No statistical method was used to predetermine sample size for the in vitro experiments, but all in vitro experiments were repeated three times.
Data exclusions	No data were excluded.
Replication	The reproducibility for each analysis was confirmed by three independent experiments.
Randomization	All samples such as cells or animals were randomly allocated into experimental groups.
Blinding	No blinding in this study. All analyses were performed using quantifiable parameters (e.g. luciferase signal values for cell viability assay and in vivo tumor growth analysis, tumorsphere number value for tumorsphere formation assay, survival time values for survival curve analysis, etc), so no bias was possible.

Reporting for specific materials, systems and methods

We require information from authors about some types of materials, experimental systems and methods used in many studies. Here, indicate whether each material, system or method listed is relevant to your study. If you are not sure if a list item applies to your research, read the appropriate section before selecting a response.

Materials & experimental systems

n/a	Involved in the study	
☐	☒	Antibodies
☐	☒	Eukaryotic cell lines
☒	☐	Palaeontology
☐	☒	Animals and other organisms
☐	☒	Human research participants
☒	☐	Clinical data

Methods

n/a	Involved in the study	
☒	☐	ChIP-seq
☒	☐	Flow cytometry
☒	☐	MRI-based neuroimaging

Antibodies

Antibodies used

Primary antibodies:
SOX2 (Santa Cruz, Cat# sc-17320, Clone Y-17, Lot G1312);
SOX2 (Bethyl, Cat# A301-739A);
WISP1 (Santa Cruz, Cat# sc-25441, Clone H-55, Lot C2312);
WISP1 (Santa Cruz, Cat# sc-133126, Clone A-9, Lot G2810);
p-Akt (Ser473) (Cell Signaling, Cat# 4058, Clone 193H12, Lot 30);
Akt (Cell Signaling, Cat# 2966, Clone 5G3, Lot 5);
Integrin α6 (Abcam, Cat# Ab181551, Clone EPR18124, Lot GR3174340-10);
Integrin α6 (Santa Cruz, Cat# sc-13542, Clone BQ16, Lot J1218);
Integrin α6 (Millipore, Cat# MAB1378, clone NKI-GoH3, Lot 2891474);
Integrin β1 (Abcam, Cat# Ab52971,Clone EP1041Y, Lot GR3230562-1);
Integrin β1 (Abcam, Cat# Ab30388, Clone JB1B, Lot GR3218801-2);
Integrin β1 (Millipore, Cat# MAB2253, Clone 6S6, Lot 3231933);
Integrin α3 (Millipore, Cat# MAB1952Z, Clone P1B5, Lot 2830819);
Integrin α7 (BIO-RAD, Cat# MCA5238Z, Clone 8G2);
Integrin β4 (Millipore, Cat# MAB2059Z, Clone ASC-8, Lot 3140199);

Active β-Catenin (Cell Signaling, Cat# 4270S, Lot 2);
β-Catenin (Cell Signaling, Cat# 9582T, Clone 6B3, Lot 5);
Tubulin (Sigma-Aldrich, Cat# 6199, Clone DM1A, Lot 116M4802V);
GAPDH (R&D Systems, Cat# MAB5718, Clone 686613, Lot CEZS0319061);
OLIG2 (R&D Systems, Cat# AF2418, , Lot UPA0617051);
Ki67 (Cell Signaling, Cat# 9129, Clone D3B5, Lot 3);
Cleaved Caspase-3 (Cell Signaling, Cat# 9661, Lot 45);
Iba1 (Abcam, Cat# Ab5076, Lot GR3175346-1);
Iba1 (Wako Chemicals, Cat# 019-19741, Lot CAQ2865);
CD11b (BIO-RAD, Cat# MCA711GT, Clone 5C6, Lot 1709);
CD163 (Thermo fisher, Cat# 14-1631-80, Clone TNKUPJ, Lot 2045378);
CD163 (Santa Cruz, Cat# sc-20066, Clone GHI/61, Lot C2619);
CD163 (BIO-RAD, Cat# MCA1853, Clone EDHu-1, Lot 147756);
CD206 (Abcam, Cat# Ab64693, Lot GR3247356-1);
CD206 (Santa Cruz, Cat# sc-58987, Clone MR5D3, Lot J1711);
CD206 (BIO-RAD, Cat# MCA2235, Clone MR5D3, Lot 148572);
Arg-1 (BD Biosciences, Cat# 610708, Clone 19, Lot 11983);
Fizz1 (Abcam, Cat# Ab39626, Lot GR12821-68);
CD11c (Thermo Fisher, Cat# PA5-35326, Lot VA2931079);
CD11c (BD Biosciences, Cat# 558079, Clone HL3, Lot 7223542);
CD16/32 (BD Biosciences, Cat# 553141, Clone 2.4G2, Lot 7152976);
iNOS (Abcam, Cat# Ab3523, Lot GR3251603-3);
iNOS (BD Biosciences, Cat# 610328, Clone 6, Lot 7208759);
MHC II (Abcam, Cat# Ab20181, Clone TAL 1B5, Lot GR3217262-1);
MHCII (Thermo Fisher, Cat# 14-5321-82, Clone M5/114.15.2, Lot 2092025);
GLUT1 (Thermo Fisher, Cat# PA1-37782, Lot OC1680971);
PE-conjugated anti-CD133 antibody (Miltenyi Biotec, Cat# 130-098-826, Clone AC133);
FITC-conjugated anti-CD15 antibody (BD Biosciences, Cat# 347423, Clone MMA).

Secondary antibodies:
Anti-rabbit IgG, HRP-linked Antibody (Cell Signaling, Cat# 7074S, Lot 26);
Anti-mouse IgG, HRP-linked Antibody (Cell Signaling, Cat# 7076S, Lot 34);
Anti-Rat IgG, HRP-linked Antibody (Cell Signaling, Cat# 7077S, Lot 9);
Anti-Goat IgG, HRP-linked Antibody (Santa Cruz, Cat# sc-2354, Lot G1217);
Donkey anti-Mouse IgG (H+L) Secondary Antibody, Alexa Fluor 488 (Thermo Fisher, Cat# A-21202, Lot 2018296);
Donkey anti-Rabbit IgG (H+L) Secondary Antibody, Alexa Fluor 488 (Thermo Fisher, Cat# A-21206, Lot 2156521);
Donkey anti-Goat IgG (H+L) Secondary Antibody, Alexa Fluor 488 (Thermo Fisher, Cat# A-11055, Lot 2059218);
Donkey anti-Rat IgG (H+L) Secondary Antibody, Alexa Fluor 488 (Thermo Fisher, Cat# A-21208, Lot 2063330);
Donkey anti-Mouse IgG (H+L) Secondary Antibody, Alexa Fluor 568 (Thermo Fisher, Cat# A-10037, Lot 2026157);
Donkey anti-Rabbit IgG (H+L) Secondary Antibody, Alexa Fluor 568 (Thermo Fisher, Cat# A-10042, Lot 2044343);
Donkey anti-Goat IgG (H+L) Secondary Antibody, Alexa Fluor 568 (Thermo Fisher, Cat# A-11057, Lot 2044862);
Donkey anti-Rat IgG (H+L) Secondary Antibody, Alexa Fluor 594 (Thermo Fisher, Cat# A-21209, Lot 2078918).

Validation

CD206 (Santa Cruz, Cat# sc-58987, Clone MR5D3, Lot J1711) is used in some publications and is validated by our lab. All other primary antibodies used in this study were validated by manufacturers and are frequently used in publications. Validation statement for each antibody is provided on the manufacturer's website. All secondary antibodies are used in many publications. The detailed information are listed as follows:

Primary antibodies:

CD206 (Santa Cruz, Cat# sc-58987, Clone MR5D3, Lot J1711):
Validation by immunofluorescence staining of glioblastoma (GBM) xenograft tissues labeling CD206 and macrophage marker (CD11b, CD163, Arg1, Fizzl, CD11c, CD16/32, iNOS or MHC II) showing CD206 is colocalized with these markers in GBM xenograft tissues.
Citation from manufacturer are listed at: https://datasheets.scbt.com/sc-58987.pdf

SOX2 (Santa Cruz, Cat# sc-17320, Clone Y-17, Lot G1312):
Validation by western blot analysis of Sox-2 expression in human and mouse embryonic stem cell lysates.
Validation by immunofluorescence staining of paraformaldehyde-fixed mouse embryonic stem cells showing nuclear localization.
Citation from manufacturer are listed at: http://datasheets.scbt.com/sc-17320.pdf

SOX2 (Bethyl, Cat# A301-739A):
Validation by western blot analysis of Sox2 expression in human H520 and HEK293T cell lysates.
Validation by immunoprecipitation of Sox2 in human H520 cell lysates.

Citation from manufacturer are listed at: https://www.bethyl.com/product/A301-739A/SOX2+Antibody#

WISP1 (Santa Cruz, Cat# sc-25441, Clone H-55, Lot C2312):
Validation by western blot analysis of human recombinant WISP-1.
Validation by Immunofluorescence staining of formalin-fixed human Hep G2 cells showing cytoplasmic localization.
Validation by Immunoperoxidase staining of formalin fixed, paraffin-embedded human colon tumor tissue showing extracellular localization.
Citation from manufacturer are listed at: http://datasheets.scbt.com/sc-25441.pdf

WISP1 (Santa Cruz, Cat# sc-133126, Clone A-9, Lot G2810):
Validation by western blot analysis of WISP-1 expression in human K-562 , Caco-2, Hep G2 and HeLa whole cell lysates.
Validation by immunofluorescence staining of formalin-fixed human Hep G2 cells showing cytoplasmic localization.
Citation from manufacturer are listed at: https://datasheets.scbt.com/sc-133126.pdf

p-Akt (Ser473) (Cell Signaling, Cat# 4058, Clone 193H12, Lot 30):
Validation by western blot analysis of extracts from untreated or PDGF-treated mouse NIH/3T3 cells, pretreated with wortmannin and/or rapamycin.
Validation by immunofluorescence staining of mouse C2C12 cells showing nuclear and cytoplasmic localization.
Citation from manufacturer are listed at: https://www.cellsignal.com/products/primary-antibodies/phospho-akt-ser473-193h12-rabbit-mab/4058

Akt (Cell Signaling, Cat# 2966, Clone 5G3, Lot 5):
Validation by immunoprecipitation of Akt from PDGF (100 ng/ml for 11 minutes) treated and untreated mouse NIH/3T3 cell lysates.
Validation by immunofluorescence staining of mouse C2C12 cells showing nuclear and cytoplasmic localization.
Citation from manufacturer are listed at: https://www.cellsignal.com/products/primary-antibodies/akt-5g3-mouse-mab/2966

Integrin α6 (Abcam, Cat# Ab181551, Clone EPR18124, Lot GR3174340-10):
Validation by immunohistochemical analysis of paraffin-embedded Human breast tissue labeling Intergrin alpha 6 showing membrane and cytoplasm staining on epithelial cells of Human breast tissue.
Validation by western blot analysis of extracts from human SW480 cells, human A549 cells, Mouse skin and Rat skin.
Citation from manufacturer are listed at: https://www.abcam.com/integrin-alpha-6-antibody-epr18124-ab181551.html

Integrin α6 (Santa Cruz, Cat# sc-13542, Clone BQ16, Lot J1218):
Validation by western blot analysis of Integrin α6 expression in human Hep G2 whole cell lysate.
Validation by immunofluorescence staining of Integrin α6 in methanol-fixed human Hep G2 cells showing membrane localization.
Citation from manufacturer are listed at: https://www.scbt.com/p/integrin-alpha6-antibody-bq16

Integrin α6 (Millipore, Cat# MAB1378, clone NKI-GoH3, Lot 2891474):
Validation by immunohistochemical staining of Integrin α6 in human intestine.
Citation from manufacturer are listed at: https://www.emdmillipore.com/US/en/product/Anti-Integrin-6-Antibody-clone-NKI-GoH3,MM_NF-MAB1378?ReferrerURL=https%3A%2F%2Fwww.google.com%2F&bd=1

Integrin β1 (Abcam, Cat# Ab52971,Clone EP1041Y, Lot GR3230562-1):
Validation by western blot analysis of Integrin β1 expression in human HeLa, HT 1080 and U2OS cell lysates.
Validation by western blot analysis of Integrin β1 expression in human U87-MG and U937 cell lysates.
Citation from manufacturer are listed at: https://www.abcam.com/integrin-beta-1-antibody-ep1041y-ab52971.html

Integrin β1 (Abcam, Cat# Ab30388, Clone JB1B, Lot GR3218801-2):
Validation by immunohistochemical staining of Integrin beta 1 in human and pig skin tissue showing membrane localization.
Citation from manufacturer are listed at: https://www.abcam.com/integrin-beta-1-antibody-jb1b-ab30388.html

Integrin β1 (Millipore, Cat# MAB2253, Clone 6S6, Lot 3231933):
Validation by immunofluorescence staining of Integrin β1 in human A431 showing membrane localization.
Citation from manufacturer are listed at: https://www.emdmillipore.com/US/en/product/Anti-Integrin-1-Antibody-clone-6S6,MM_NF-MAB2253#overview

Integrin α3 (Millipore, Cat# MAB1952Z, Clone P1B5, Lot 2830819):
Validation by immunofluorescence staining of Integrin α3 in human A431 cells showing membrane localization.
Validation by cell surface staining of Integrin α3 in human HT-1080 cells via Flow Cytometry analysis.
Citation from manufacturer are listed at: https://www.emdmillipore.com/US/en/product/Anti-Integrin-3-Antibody-clone-P1B5-azide-free,MM_NF-MAB1952Z

Integrin α7 (BIO-RAD, Cat# MCA5238Z, Clone 8G2):
Validation by western Blot analysis of integrin α7 expression in human Jurkat cell lysate.
Citation from manufacturer are listed at: https://www.bio-rad-antibodies.com/monoclonal/human-integrin-alpha-7-

antibody-8g2-mca5238.html?f=preservative%20free

Integrin β4 (Millipore, Cat# MAB2059Z, Clone ASC-8, Lot 3140199):
Validation by Flow cytometric analysis of Integrin β4 in human Hela and A431 cells.
Citation from manufacturer are listed at: https://www.emdmillipore.com/US/en/product/Anti-Integrin-4-Antibody-clone-ASC-8-Azide-Free,MM_NF-MAB2059Z?ReferrerURL=https%3A%2F%2Fwww.google.com%2F&bd=1#overview

Active β-Catenin (Cell Signaling, Cat# 4270S, Lot 2):
Validation by western blot analysis of Active β-Catenin in total human SW480 cell lysates.
Validation by immunoprecipitation of Active β-Catenin in total human SW480 cell lysates.
Citation from manufacturer are listed at: https://www.cellsignal.com/products/primary-antibodies/non-phospho-active-b-catenin-ser33-37-thr41-antibody/4270

β-Catenin (Cell Signaling, Cat# 9582T, Clone 6B3, Lot 5):
Validation by western blot analysis of β-Catenin in total cell lysates from human 293, Rat Rat2 and Monkey COS cells.
Immunohistochemical analysis of β-Catenin in paraffin-embedded human hepatocellular carcinoma, human lung carcinoma.
Citation from manufacturer are listed at: https://www.cellsignal.com/products/primary-antibodies/b-catenin-6b3-rabbit-mab/9582

Tubulin (Sigma-Aldrich, Cat# 6199, Clone DM1A, Lot 116M4802V);
Validation by western blot analysis of α-Tubulin in total cell lysates from human HeLa, JURKAT, Monkey COS7, mouse NIH-3T3, Rat PC-12, RAT2, Chinese hamster CHO, bovine MDBK and dog MDCK cells.
Validation by immunofluorescence staining of α-Tubulin in Lamin A/C knockout mice embryo fibroblasts.
Citation from manufacturer are listed at: https://www.sigmaaldrich.com/catalog/product/sigma/t6199?lang=en&region=US

GAPDH (R&D Systems, Cat# MAB5718, Clone 686613, Lot CEZS0319061):
Validation by western blot analysis of GAPDH expression in lysates from human brain tissue, mouse brain tissue, and rat brain tissue.
Validation by immunofluorescence staining of GAPDH in immersion fixed human HeLa cells.
Citation from manufacturer are listed at: https://www.rndsystems.com/products/human-mouse-rat-gapdh-antibody-686613_mab5718

OLIG2 (R&D Systems, Cat# AF2418, , Lot UPA0617051):
Validation by immunoprecipitation of OLIG2 in lysates from A172 human glioblastoma cell line.
Validation by immunofluorescence staining of OLIG2 in differentiated rat cortical stem cells showing showing nuclear localization.
Citation from manufacturer are listed at: https://www.rndsystems.com/products/human-mouse-rat-olig2-antibody_af2418#product-details

Ki67 (Cell Signaling, Cat# 9129, Clone D3B5, Lot 3):
Validation by immunofluorescence staining of Ki67 in human Hela cells and the ventricular zone in P21 mouse brain showing nuclear localization.
Validation by Flow cytometric analysis of Ki67 in human Jurkat cells.
Citation from manufacturer are listed at: https://www.cellsignal.com/products/primary-antibodies/ki-67-d3b5-rabbit-mab/9129

Cleaved Caspase-3 (Cell Signaling, Cat# 9661, Lot 45):
Validation by western blot analysis of Cleaved Caspase-3 expression in cell lysates from human HeLa, mouse NIH/3T3 and Rat C6 cells.
Validation by immunohistochemical analysis of Cleaved Caspase-3 in paraffin-embedded human tonsil, showing cytoplasmic and perinuclear localization in apoptotic cells.
Citation from manufacturer are listed at: https://www.cellsignal.com/products/primary-antibodies/cleaved-caspase-3-asp175-antibody/9661

Iba1 (Abcam, Cat# Ab5076, Lot GR3175346-1):
Validation by immunofluorescence staining of Iba1 in the human macrophage cell line Mono-Mac 6.
Validation by western blot analysis of Iba1 in lyates from human lymph node tissue and rat brain tissue
Validation by immunohistochemical analysis of Iba1 in rat brain tissue.
Citation from manufacturer are listed at: https://www.abcam.com/iba1-antibody-ab5076.html

Iba1 (Wako Chemicals, Cat# 019-19741, Lot CAQ2865):
Validation by immunohistochemical analysis of Iba1 in Rat brain cortex.
Citation from manufacturer are listed at: https://labchem-wako.fujifilm.com/us/product/detail/W01W0101-1974.html

CD11b (BIO-RAD, Cat# MCA711GT, Clone 5C6, Lot 1709):
Validation by immunofluorescence staining of CD11b in mouse lymph node cryosection.
Validation by immunofluorescence staining of CD11b in mixed glial cultures and in the retinal outer nuclear layer in a murine model of retinitis pigmentosa.

Citation from manufacturer are listed at: https://www.bio-rad-antibodies.com/monoclonal/mouse-cd11b-antibody-5c6-mca711.html?f=purified

CD163 (Thermo fisher, Cat# 14-1631-80, Clone TNKUPJ, Lot 2045378):
Validation by immunohistochemical analysis of CD163 in mouse spleen and liver tissue.
Citation from manufacturer are listed at: https://www.thermofisher.com/antibody/product/CD163-Antibody-clone-TNKUPJ-Monoclonal/14-1631-82

CD163 (Santa Cruz, Cat# sc-20066, Clone GHI/61, Lot C2619):
Validation by western blot analysis of CD163 expression in whole cell lysate of THP-1 human monocytic cell line.
Validation by Flow cytometric analysis of CD163 in human peripheral blood leukocytes.
Citation from manufacturer are listed at: https://www.scbt.com/p/cd163-antibody-ghi-61

CD163 (BIO-RAD, Cat# MCA1853, Clone EDHu-1, Lot 147756):
Validation by immunofluorescence staining of CD163 in human tonsil tissue.
Validation by Flow cytometric analysis of CD163 on human blood mononuclear cells.
Citation from manufacturer are listed at: https://www.bio-rad-antibodies.com/monoclonal/human-cd163-antibody-edhu-1-mca1853.html?f=purified

CD206 (Abcam, Cat# Ab64693, Lot GR3247356-1):
Validation by western blot analysis of CD206 (Mannose Receptor) expression in lysates from rat Liver, lung, human Lung and mouse lung.
Validation by immunofluorescence staining of CD206 in human MOLT4 cells showing membrane localization.
Validation by immunohistochemical analysis of CD206 in human, mouse and rat lung tissues.
Citation from manufacturer are listed at: https://www.abcam.com/mannose-receptor-antibody-ab64693.html

CD206 (BIO-RAD, Cat# MCA2235, Clone MR5D3, Lot 148572):
Validation by immunoperoxidase staining of CD206 In a mouse lymph node.
Validation by immunofluorescence staining of CD206 in a mouse lymph node.
Citation from manufacturer are listed at: https://www.bio-rad-antibodies.com/monoclonal/mouse-cd206-antibody-mr5d3-mca2235.html?f=purified

Arg-1 (BD Biosciences, Cat# 610708, Clone 19, Lot 11983):
Validation by western blot analysis of Arg-1 expression in lysate from mouse liver.
Validation by immunofluorescence staining of Arg-1 in mouse macrophages.
Citation from manufacturer are listed at: https://www.bdbiosciences.com/eu/reagents/research/antibodies-buffers/cell-biology-reagents/cell-biology-antibodies/purified-mouse-anti-arginase-i-19arginase-i/p/610708

Fizz1 (Abcam, Cat# Ab39626, Lot GR12821-68):
Validation by immunohistochemical analysis of Fizz1 (RELM alpha) in mouse liver and cecum tissue.
Citation from manufacturer are listed at: https://www.abcam.com/relm-alpharetnla-antibody-ab39626.html

CD11c (Thermo Fisher, Cat# PA5-35326, Lot VA2931079):
Validation by western blot analysis of CD11c in lysate from human MDA-MAB231 cells
Validation by immunofluorescence staining of CD11c in human hepatocarcinoma tissue.
Validation by immunohistochemical staining of CD11c in human hepatocarcinoma tissue.
Validation by Flow cytometric analysis of CD11c on human SK-BR-3 cells.

CD11c (BD Biosciences, Cat# 558079, Clone HL3, Lot 7223542):
Validation by Flow cytometric analysis of CD11c on mouse splenocytes.
Citation from manufacturer are listed at: https://www.bdbiosciences.com/eu/reagents/research/antibodies-buffers/immunology-reagents/anti-mouse-antibodies/cell-surface-antigens/pe-cy7-hamster-anti-mouse-cd11c-hl3/p/558079

CD16/32 (BD Biosciences, Cat# 553141, Clone 2.4G2, Lot 7152976):
Validation by Flow cytometric analysis of CD16/32 on mouse spleen cells.
Citation from manufacturer are listed at: https://www.bdbiosciences.com/us/applications/research/b-cell-research/surface-markers/mouse/purified-rat-anti-mouse-cd16cd32-mouse-bd-fc-block-24g2/p/553141

iNOS (Abcam, Cat# Ab3523, Lot GR3251603-3):
Validation by immunofluorescence staining of iNOS in human A549 and mouse NIH-3T3 cells.
Validation by immunohistochemical staining of iNOS in human heart tissue.
Citation from manufacturer are listed at: https://www.abcam.com/inos-antibody-ab3523.html

iNOS (BD Biosciences, Cat# 610328, Clone 6, Lot 7208759):
Validation by western blot analysis of iNOS expression in lysate from mouse RAW 264.7 macrophages.
Validation by immunofluorescence staining of iNOS in mouse macrophages stimulated with 10 ng/mL IFNγ and 1 μg/mL LPS.

Citation from manufacturer are listed at: https://www.bdbiosciences.com/us/reagents/research/antibodies-buffers/cell-biology-reagents/cell-biology-antibodies/purified-mouse-anti-inosnos-type-ii-6inosnos-type-ii/p/610328

MHC II (Abcam, Cat# Ab20181, Clone TAL 1B5, Lot GR3217262-1):
Validation by western blot analysis of MHC II expression in lysate from human spleen, tonsil, liver and colon tissues.
Validation by immunohistochemical staining of MHC II in human normal thymus tissue and skin melanoma.
Validation by Flow cytometric analysis of MHC II on human peripheral blood mononuclear cells.
Citation from manufacturer are listed at: https://www.abcam.com/hla-dr-antibody-tal-1b5-ab20181.html

MHCII (Thermo Fisher, Cat# 14-5321-82, Clone M5/114.15.2, Lot 2092025):
Validation by Flow cytometric analysis of MHCII on mouse splenocytes.
Citation from manufacturer are listed at: https://www.thermofisher.com/antibody/product/MHC-Class-II-I-A-I-E-Antibody-clone-M5-114-15-2-Monoclonal/14-5321-82

GLUT1 (Thermo Fisher, Cat# PA1-37782, Lot OC1680971):
Validation by immunohistochemical staining of Glut1 in human Esophagous Cancer Tissue.
Citation from manufacturer are listed at: https://www.fishersci.com/order/genome-database/generatePdf?productName=GLUT1&assayType=PRANT&detailed=true&productId=PA1-37782

PE-conjugated anti-CD133 antibody (Miltenyi Biotec, Cat# 130-098-826, Clone AC133):
Validation by Flow cytometric analysis of CD133 on human peripheral blood mononuclear cells (PBMCs).
Citation from manufacturer are listed at: http://ulab360.com/files/prod/manuals/201604/15/596802001.pdf

FITC-conjugated anti-CD15 antibody (BD Biosciences, Cat# 347423, Clone MMA).
Validation by Flow cytometric analysis of CD15 on human whole blood.
Citation from manufacturer are listed at: https://www.bdbiosciences.com/ds/is/tds/23-1319.pdf

Secondary antibodies:

Anti-rabbit IgG, HRP-linked Antibody (Cell Signaling, Cat# 7074S, Lot 26):
Citation from manufacturer are listed at: https://www.cellsignal.com/products/secondary-antibodies/anti-rabbit-igg-hrp-linked-antibody/7074

Anti-mouse IgG, HRP-linked Antibody (Cell Signaling, Cat# 7076S, Lot 34):
Citation from manufacturer are listed at: https://www.cellsignal.com/products/secondary-antibodies/anti-mouse-igg-hrp-linked-antibody/7076

Anti-Rat IgG, HRP-linked Antibody (Cell Signaling, Cat# 7077S, Lot 9):
Citation from manufacturer are listed at: https://www.cellsignal.com/products/secondary-antibodies/anti-rat-igg-hrp-linked-antibody/7077

Anti-Goat IgG, HRP-linked Antibody (Santa Cruz, Cat# sc-2354, Lot G1217):
Citation from manufacturer are listed at: https://www.scbt.com/p/mouse-anti-goat-igg-hrp

Donkey anti-Mouse IgG (H+L) Secondary Antibody, Alexa Fluor 488 (Thermo Fisher, Cat# A-21202, Lot 2018296):
Citation from manufacturer are listed at: https://www.thermofisher.com/antibody/product/Donkey-anti-Mouse-IgG-H-L-Highly-Cross-Adsorbed-Secondary-Antibody-Polyclonal/A-21202

Donkey anti-Rabbit IgG (H+L) Secondary Antibody, Alexa Fluor 488 (Thermo Fisher, Cat# A-21206, Lot 2156521):
Citation from manufacturer are listed at: https://www.thermofisher.com/antibody/product/Donkey-anti-Rabbit-IgG-H-L-Highly-Cross-Adsorbed-Secondary-Antibody-Polyclonal/A-21206

Donkey anti-Goat IgG (H+L) Secondary Antibody, Alexa Fluor 488 (Thermo Fisher, Cat# A-11055, Lot 2059218):
Citation from manufacturer are listed at:https://www.thermofisher.com/antibody/product/Donkey-anti-Goat-IgG-H-L-Cross-Adsorbed-Secondary-Antibody-Polyclonal/A-11055

Donkey anti-Rat IgG (H+L) Secondary Antibody, Alexa Fluor 488 (Thermo Fisher, Cat# A-21208, Lot 2063330):
Citation from manufacturer are listed at: https://www.thermofisher.com/antibody/product/Donkey-anti-Rat-IgG-H-L-Highly-Cross-Adsorbed-Secondary-Antibody-Polyclonal/A-21208

Donkey anti-Mouse IgG (H+L) Secondary Antibody, Alexa Fluor 568 (Thermo Fisher, Cat# A-10037, Lot 2026157):
Citation from manufacturer are listed at: https://www.thermofisher.com/antibody/product/Donkey-anti-Mouse-IgG-H-L-Highly-Cross-Adsorbed-Secondary-Antibody-Polyclonal/A10037

Donkey anti-Rabbit IgG (H+L) Secondary Antibody, Alexa Fluor 568 (Thermo Fisher, Cat# A-10042, Lot 2044343):

	Citation from manufacturer are listed at: https://www.thermofisher.com/antibody/product/Donkey-anti-Rabbit-IgG-H-L-Highly-Cross-Adsorbed-Secondary-Antibody-Polyclonal/A10042<br><br>Donkey anti-Goat IgG (H+L) Secondary Antibody, Alexa Fluor 568 (Thermo Fisher, Cat# A-11057, Lot 2044862):<br>Citation from manufacturer are listed at:https://www.thermofisher.com/antibody/product/Donkey-anti-Goat-IgG-H-L-Cross-Adsorbed-Secondary-Antibody-Polyclonal/A-11057<br><br>Donkey anti-Rat IgG (H+L) Secondary Antibody, Alexa Fluor 594 (Thermo Fisher, Cat# A-21209, Lot 2078918):<br>Citation from manufacturer are listed at: https://www.thermofisher.com/antibody/product/Donkey-anti-Rat-IgG-H-L-Highly-Cross-Adsorbed-Secondary-Antibody-Polyclonal/A-21209

Eukaryotic cell lines

Policy information about cell lines

Cell line source(s)	Glioma stem cells (GSCs) and Non-stem tumor cells (NSTCs) were isolated from primary GBM tumors or GBM xenografts and functionally characterized by our lab. U937 cells were obtained from ATCC.
Authentication	We used STR genetic analyses to characterize the GSCs and NSTCs derived from human primary GBMs. We maintained these GSC lines as xenografts in immunocompromised mice. Human cell authentication utilizing STR profiling for GSCs, NSTCs and other cells (U937) were performed by professional services provided by ATCC and Duke Medical Center.
Mycoplasma contamination	All cell lines were tested for mycoplasma contamination. All cell lines used in this study were negative for mycoplasma.
Commonly misidentified lines (See ICLAC register)	None of cell lines used in this study are listed by ICLAC.

Animals and other organisms

Policy information about studies involving animals; ARRIVE guidelines recommended for reporting animal research

Laboratory animals	6-7 week-old female or male NSG mice (The Jackson laboratory, 005557, or from internal breeding by our animal facility) were used. Mice were housed under a 12-h light/12-h dark cycle in a temperature (20-26°C) and humidity (30%-70%) controlled environment and were fed ad libitum.
Wild animals	No wild animals were used.
Field-collected samples	No field-collected samples were used.
Ethics oversight	All animal procedures were approved by the Institutional Animal Care and Use Committee at Cleveland Clinic.

Note that full information on the approval of the study protocol must also be provided in the manuscript.

Human research participants

Policy information about studies involving human research participants

Population characteristics	Human glioblastoma (GBM) used in this study were histopathologically and clinically diagnosed. Among these patients, there are 9 male and 2 female patients. Except for an 8-year-old patient, the age of other patients ranges from 26-76. The detailed information is listed as follows: T387 GSCs and NSTCs were derived from a GBM from a 76-year old female patient. D456 GSCs and NSTCs were derived from a GBM from an 8-year old female patient. T4121 GSCs and NSTCs were derived from a GBM from a 53-year old male patient. T3094 GSCs and NSTCs were derived from a GBM from a 63-year old male patient. T3565 GSCs and NSTCs were derived from a GBM from a 32-year old male patient. T3359 GSCs and NSTCs were derived from a GBM from a 31-year old male patient. CW1797 GBM specimens were collected from a 57-year old male patient. CW1798 GBM specimens were collected from a 47-year old male patient. CW2360 GBM specimens were collected from a 26-year old male patient. DI-74 GBM specimens were collected from a 52-year old male patient. CCF2445 GBM specimens were collected from a 50-year old male patient.
Recruitment	Human glioblastoma specimens were collected with informed consent by the Brain Tumor and Neuro-Oncology Center at Cleveland Clinic and University Hospitals of Case Western Reserve University. The selection of patients was random and we believe that there was no bias in data selection.
Ethics oversight	All procedures performed using human tissues were approved by the ethics committee of Cleveland Clinic and University Hospitals.

Note that full information on the approval of the study protocol must also be provided in the manuscript.
